# Toxicological safety of VOHO Hemp Oil; a supercritical fluid extract from the aerial parts of hemp

**DOI:** 10.1371/journal.pone.0261900

**Published:** 2021-12-31

**Authors:** Margitta Dziwenka, Laurie Dolan, Jason Mitchell

**Affiliations:** 1 GRAS Associates, LLC North Bethesda, Maryland, United States of America; 2 HempFusion, Denver, Colorado, United States of America; Goa University, India, INDIA

## Abstract

VOHO Hemp Oil (Verdant Nature LLC (in collaboration with HempFusion)) is an extract of the aerial parts of hemp (*Cannabis sativa* L) manufactured using a supercritical CO_2_ extraction process. The results of four safety studies are reported here including a bacterial reverse mutation assay, an *in vivo* mammalian micronucleus study, a maximum tolerated dose study in rats and a 90-day repeat dose subchronic toxicity study in rats. VOHO Hemp oil can contain up to 30% phytocannabinoids and less than 0.2% is tetrahydrocannabinol (THC). VOHO Hemp Oil was found to be non-mutagenic in the bacterial reverse mutation assay and was negative for inducing micronuclei in the rat bone marrow micronucleus assay. The maximum tolerated dose in male and female Wistar rats was 2250 mg/kg bw/day. A 90-day repeat dose study was conducted in male and female Wistar rats according to OECD Guideline 408 and included a 21-day recovery period. The doses used in the study were 0, 25, 90 and 324 mg/kg bw per day in the main study, and in the recovery phase a control and 324 mg/kg bw/day group were included. One mortality was reported during the study, a high dose female, and test substance-related adverse clinical signs were reported in the high dose group. Other test substance-related changes noted in the high dose group included changes in body weights, activated partial thromboplastin time (APTT) values, and in absolute and relative organ weights. Based on the results of the study, the no observed adverse effect level (NOAEL) for VOHO Hemp Oil was determined to be 90 mg/kg bw/day in both male and female Wistar rats.

## Introduction

Recently there has been a sharp increase in consumer interest in hemp extracts, however *Cannabis Sativa* L. has a long history of therapeutic uses and as an agricultural crop [1, 2]. The current interest in hemp is not limited to the abundance of phytochemicals, it is also gaining popularity as a valuable agriculture crop. The passage of the Agriculture Improvement Act of 2018 which is sometimes referred to as the 2018 Farm Bill, has also served to increase the interest in hemp products [3].

Hemp extracts contain a number of biologically active compounds including cannabinoids, terpenoids, phenolic compounds, and others, many of which are still being identified and categorized [1, 2, 4]. With the increase in consumer consumption of hemp extracts there has been an increased interest in and demand for determining the safety of these extracts. There are recently published studies in both humans and laboratory animals evaluating the safety of orally consumed hemp extracts, however with the variation in the composition of these extracts, comparison of the information must be conducted in tandem with a detailed evaluation of the extract composition [5–7]. The bioactivity or potential for hemp extracts to cause toxicity may be influenced by method of manufacture or slight differences in the chemical profile. Until the toxicity of various hemp extracts can be accurately predicted from the chemical profile, the safety of hemp extracts should be determined on an individual basis.

The results of a bacterial reverse mutation assay, and an *in vivo* micronucleus study, maximum tolerated dose study and a 90-day repeat dose toxicity study in rats are reported here for a supercritical fluid extract produced from the aerial parts of Hemp (VOHO Hemp Oil).

## Materials and methods

### Test substance

The test substance, referred to as VOHO Hemp Oil (Verdant Nature LLC, Warsaw, Poland) (in collaboration with HempFusion) is a Cannabis sativa L (hemp) extract manufactured using supercritical CO_2_ extraction. VOHO Hemp oil may contain 10–30% cannabinoids (of which THC is <0.2%), 1–3% terpenoids, 70–85% fatty acids, 1–5% sterols and 1–5% tocopherol/tocotrienols. The lot used in the 90-day study contained 28.14% cannabinoids, of which 25.2% was cannabidiol (CBD).

For the bacterial reverse mutation studies, the test material was formulated as a solution in dimethylsulfoxide (DMSO, Sigma-Aldrich, lot no BCBV2983) to provide dose levels of up to 5000 μg/mL. For the *in vivo* micronucleus, maximum tolerated dose and 90-day repeat dose studies, the test material was prepared in hemp seed oil (Verdant Oasis LLC, lot SC19G02-1) by mixing the appropriate amount of the test material into the appropriate amount of vehicle. Hemp seed oil is a suitable vehicle for hemp extracts and has been approved for addition to foods and animal feeds in multiple countries [8]. In addition, a GRAS Notification (GRN 000778) for Hemp Seed Oil received a “no questions” response from FDA [9]. The formulated dosing solutions were prepared weekly and stored at 2–8°C and were stable for up to 7 days. Prior to dosing, the test material formulations were brought to room temperature and thoroughly mixed.

For the 90-day study, the concentrations of the extract in the dose solutions were analyzed at the initiation of the study, during the 6^th^ week of the study and then again at the end of the study. The stability of the test material in the vehicle was also confirmed. Results of analytical testing for the 90-day studies showed that the test material preparations were in the range of 80–120% of the target concentrations throughout the study.

### Guidelines

The bacterial reverse mutation assay was conducted in compliance with Good Laboratory Practices (GLPs) [10]. The mammalian erythrocyte micronucleus assay, maximum tolerated and the 90-day toxicity studies were also conducted in compliance with the following GLPs: OECD Principles of Good Laboratory Practices and EU Directive 2004/10/EC as well as local regulations [10, 11].

The bacterial reverse mutation study was performed according to OECD Guideline for the Testing of Chemicals No. 471 (1997) and the Redbook 2000, Chapter IV.C.1.a Bacterial Reverse Mutation Test (2018) but was performed as an Ames fluctuation assay which is not described in detail in these guidelines [12, 13]. The assay is based on the same principle as the traditional test but uses liquid media, a microplate format as well as a colorimetric method rather than traditional counting of colonies. The mammalian erythrocyte micronucleus assay was performed according to the OECD Guideline for Testing of Chemicals No. 474: “Mammalian Erythrocyte Micronucleus Test” and Redbook 2000, Chapter IV.C.1.d Mammalian Erythrocyte Micronucleus Test [13, 14]. The maximum tolerated dose study in rats was conducted according to ICH Guideline M3(R2), OECD Guidance Document on Acute Oral Toxicity Testing and Redbook IV.C. 2. Acute Oral Toxicity Tests [15–17]. The 90-day repeat dose study in rats was conducted according to OECD Guideline 408, Redbook IV.C. 4. a. and EU Method B.26; Council Regulation (EC) No. 440/2008 [13, 18, 19]. All studies were conducted at the Lukasiewicz Research Network–Institute of Industrial Organic Chemistry, Branch Pszczyna in Pszczyna, Poland.

The maximum tolerated dose and 90-day repeat dose studies were conducted with the approval of the Local Ethical Committee for Animal Experimentation in Katowice–Resolution No. 58/2019.

### Bacterial reverse mutation assay

The Ames fluctuation assay was used to evaluate the mutagenic potential of the test material and is based on the same principle as the traditional test. The Microplate format (MPF) of the study was conducted using an Ames MPF Penta I kit from Xenometrix (Gewerbesrasse 25, CH-4123) (Xenometrix, 2021). Bacterial strains utilized in all experiments were provided by Xenometrix and included *S*. *typhimurium* tester strains TA98, TA100, TA1535 and TA1537 and mixed strains of *E*. *coli*; WP2 uvrA and WP2 pKM101. The study was performed in the absence and presence of a metabolic activation system (S9 mix) prepared from the liver of a male Sprague Dawley rat induced with phenobarbital/β-naphthoflavone (Xenometrix). Positive controls were utilized in the study. The positive controls used in the absence of S9 mix were 2-nitrofluorene for *S*. *typhimurium* TA98, 4-nitroquinoline N-oxide for TA100 and *E*. *coli* WP2 (mix), N-4-aminocitidine for TA1535 and 9-aminoacridine for TA1537. The positive control for all bacterial strains in the presence of S9 mix was 2-aminoanthracene (2-AA). DMSO was used as the negative control. A preliminary study was conducted to determine the potential cytotoxicity of the test material and the highest concentration to be used in the main study. To prepare the 24-well exposure plates, a stock solution was prepared and from there five concentrations of the test material were prepared with a 3.16-fold decrease in concentration. The solutions were prepared shortly before the experiment and three replicates were used for each concentration: 0, 16, 50, 159, 500, 1582 and 5000 μg/mL. The previously prepared overnight cultures were mixed with suitable exposure medium and the S9 fraction was added to one set of each strain. The bacteria were transferred to the 24-well plate containing the test material solutions and the plate placed onto a shaker plate for 90 minutes at 120 rpm and 37±1°C. Following the incubation period, the plates were removed, and suitable indicator medium was added to each well. The bacterial suspension from one well of the 24-well plate was transferred to 48 wells of a 384-well plate which was then placed into a plastic bag in a 37±1°C dry incubator for 48±4 hours. The plates were removed from the incubator and the numbers of positive wells were visually counted. The mean number and standard deviation of the positive wells per concentration were calculated. The binomial B-value (B≥0.99) was used to determine statistical significance between test material or positive or negative control. The fold induction over the baseline or ratio of the mean number of positive wells for the concentration divided by the baseline was calculated. Baseline was obtained by adding one standard deviation to the main number of positive wells of the negative control from the current test and from historical data as well as from the maximum acceptable value for the strain. The test results were considered valid if all of the following criteria were satisfied; the OD600 should be at least 2.0 for the overnight cultures and ≤0.05 for the negative control; the results of the positive and negative controls should be within a specified range and the number of positive wells for the positive control should be within historical range.

### Mammalian erythrocyte micronucleus study

The study was conducted to evaluate the potential genotoxicity of the test material in an *in vivo* micronucleus assay. Male and female Wistar rats (n = 30/sex) were used and divided into six groups: one negative control, two positive controls and three treatment groups (65.2, 250 and 1000 mg/kg body weight (bw)/day). The positive controls used in the study were cyclophosphamide (20 mg/kg bw/day) and mitomycin (2.5 mg/kg bw/day) and the negative/vehicle control was hemp seed oil (Verdant Oasis, LLC, lot SC19G02-1). Animals were dosed orally three times in a 24-hour interval with the exception that mitomycin was administered intraperitoneally rather than orally. Animals were observed daily for mortality or clinical signs of toxicity. All animals were weighed at the start of the study and at euthanasia. At 18 and 24 hours after the last dose, all animals were euthanized, and bone marrow was collected and suspended in fetal bovine serum. Bone marrow smears were prepared 30 minutes later, stained with the May-Grunwald-Giemsa method, and evaluated microscopically. The criteria for the identification of the micronuclei were as follows; micronuclei were identified as darkly stained and generally round (although almond and ring shaped are occasionally seen), sharp bordered and generally between 1/20^th^ and 1/5^th^ the size of the polychromatic erythrocytes (PCE). The following indices were scored; the PCE ratio for each animal by counting a total of at least 200 erythrocytes, at least 2000 immature erythrocytes per animal were scored for the incidence of micronucleated immature erythrocytes and the frequency of the polychromatic erythrocytes containing micronuclei (MNPCE) were calculated and the proportion of immature erythrocytes to total erythrocytes in treated animals were higher than 20%. The genotoxic index was expressed as frequency of MNPCE in 2000 PCE per animal and cytotoxicity was expressed as a PCE ratio. Three doses of the test material were analyzed to obtain dose response information. The MNPCE frequency as well as the PCE ratio in the treated groups was compared with the negative control group with males and females being analyzed separately; the micronucleated erythrocyte frequency as well as the PCE ratio in the positive control groups was compared to the negative control group separately for males and females.

### Maximum tolerated dose study

A single dose maximum tolerated dose (MTD) study was conducted to evaluate the potential *in vivo* toxicity of the test material. The first dose utilized was 1000 mg/kg bw and the study was conducted as a typical MTD study where the second dose was selected based on the results of the first dose, the third dose is based on the results of the second dose, etc. Male and female Wistar rats (Cmdb: Wi; outbred) were used in this study (n = 30/sex) and were approximately 8 weeks of age at the start of the study. Animals were divided into six groups (5/sex), one of which was a vehicle control group that received hemp seed oil. Animals were fasted for 15 hours prior to dosing and food was returned three hours after dosing. Animals were dosed with the test substance based on their body weight taken just prior to dosing. The test material was prepared just prior to dosing by dissolving the test material in hemp seed oil at the appropriate concentration. Animals were then dosed via gavage at a volume of 0.4 mL/100 g bw. Doses administered (in order) were 1000 mg/kg bw, 2000 mg/kg bw, 3000 mg/kg bw, 2500 mg/kg bw and 2250 mg/kg bw. The decision to include a group dosed with 2250 mg/kg bw was based on the results of dosing with higher doses (see Results Section). Following dosing, animals were observed twice daily for morbidity or mortality. In addition, detailed clinical observations were conducted prior to dosing on dose day and then at 10- and 30-minutes following dosing, as well as at 1, 2, 3, 4 and 5-hours following dosing. Detailed clinical examinations were then conducted on each animal once daily for an additional 13 days. Body weights were taken on day 0 (prior to dosing), and then on study day 1, 2, 3, 4, 5, 7 and 13 and on day 14 after fasting prior to euthanasia. Food was weighed on study day 3, 7 and 13 and food intake/100 g bw/day was determined. Animals were fasted overnight prior to euthanasia on study day 14. Animals were anesthetized with a xylazine-ketamine mixture and blood collected from each animal. Blood was collected for hematological, biochemical and coagulation evaluation. The hematological parameters evaluated included leukocyte count, erythrocyte count, thrombocyte count, hemoglobin level, hematocrit, mean corpuscular volume, mean corpuscular hemoglobin weight, and mean corpuscular hemoglobin concentration (MCHC). The coagulation parameters evaluated included prothrombin time (PTT) and activated partial thromboplastin time (APTT). The following parameters were evaluated; total protein, albumin, globulin, total cholesterol, urea nitrogen, creatinine, fasting glucose, total bilirubin, bile acids, calcium, sodium, potassium, chloride, inorganic phosphorus, aspartate aminotransferase (AST), alanine aminotransferase (ALT), and alkaline phosphatase (ALP). Following blood collection, animals were euthanized with an overdose of xylazine-ketamine and then subjected to a gross post-mortem examination which included observations of the external surfaces and orifices, as well as the cranial, thoracic, and abdominal cavities and their contents. No tissues were preserved.

### 90-day oral repeat dose study

Wistar rats (Cmdb: Wi; outbred) were used in the 90-day study and were approximately 8 weeks old at the start of the experiment. Animals were acclimated for at least 5 days prior to testing. Rats were housed in plastic bottom cages with wire lids containing wood chip bedding and environmental enrichment. Environmental enrichment, such as tubes, huts and wooden chew blocks, are added to the cage for laboratory animals to ensure their wellbeing, allow for species typical behaviors and improve research results [20]. Animal rooms housed in environmental controlled rooms with a 12-hour light/dark cycle and 15 air changes per hour. The temperature was maintained between 19.5 and 24.0°C and the relative humidity between 26.5 and 64.5%. Animals were housed individually and supplied *ad libitum* access to food (Altromin 1324 P TPF) and tap water. Animals were divided into six groups according to stratification by body weight (n = 10/sex/group); a control group receiving hemp seed oil only, and three treatment groups receiving either 25, 90 or 324 mg of test material/kg bw/day and two recovery groups. The two recovery groups included in the study received either the hemp seed oil or the high dose of 324 mg/kg bw/day for 90 days but then were observed for an additional 21 days following the end of dosing to evaluate reversibility, persistence, or delayed occurrence of potential toxic changes. All animals were dosed orally with either the control or test material using a stomach tube, once daily for 90 consecutive days. The first day of dosing was considered study day 0. The animals were given a constant volume of 0.4 mL/100 g bw calculated on the latest body weight taken.

During the study, animals were observed twice daily for mortality and morbidity and detailed clinical observations were conducted before dosing on day 0 and then once weekly thereafter. The detailed clinical observations included, but were not limited to, changes in the skin, fur, eyes, mucous membranes as well as changes in the respiratory, circulatory, autonomic, and central nervous systems, somatic activity, and behavior. Ophthalmological examinations were performed on all animals prior to the start of dosing and then again on the 89^th^ day for the main study animals and the 110^th^ day for the recovery groups. Body weights were taken prior to dosing on day 0 and then twice weekly during the study and on the final day of the study and food intake was determined once weekly during the study. During the 13^th^ week of the study, all animals were subjected to a behavioral evaluation/functional observational battery (FOB) and the recovery animals were subjected to a second evaluation during the sixteenth week of the study. The behavioral measurements evaluated included behavior in an open field such as involuntary tonic and clonic movements and their type, changes in gait, arousal and stereotypical behavior, number of fecal pellets and urine pools. In addition, responses to stimuli, fore- and hindlimb grip strength and sensorimotor activity were assessed. Locomotor activity, both vertical and horizontal, was measured using the ActiMot System (TSE systems) for 30 minutes. Horizontal activity was expressed by distance covered and vertical activity was expressed as the number of rearings.

On the day prior to euthanasia (day 89 for the main study animals and 110 for the recovery animals), animals were placed in metabolic cages with no access to food but *ad libitum* access to water, for approximately 15 hours. The following day, the fasted animals were anesthetized with a xylazine-ketamine mixture and blood samples were collected from the heart. The animals were then euthanized with an additional dose of xylazine-ketamine. Hematology parameters evaluated on all animals included evaluation of leukocyte count, erythrocyte count, thrombocyte count, hemoglobin level, hematocrit value, mean corpuscular volume, mean corpuscular hemoglobin, MCHC and reticulocyte number. Differential leukocyte evaluations were also conducted. Coagulation parameters evaluated included PTT and APTT. Biochemical parameters evaluated included total protein, albumin, globulin (calculated), total cholesterol, urea nitrogen, creatinine, glucose, bilirubin, bile acids, calcium, sodium, potassium, chlorides, inorganic phosphorus, high density lipoprotein (HDL) and low-density lipoprotein (LDL) cholesterol, AST, ALT and ALP. The levels of thyroxine (T4), triiodothyronine (T3) and thyroid stimulating hormone (TSH) were determined in the serum. The volume of urine was determined along with urine color, specific gravity, pH, protein, glucose, ketone, bilirubin, blood, urobilinogen, leukocyte count and nitrates. Urine sediment was also collected and evaluated for squamous epithelium, transitional epithelium, leukocytes, erythrocytes, bacteria, and any crystals determined. Bone marrow was obtained from the femur, slides prepared and stained with May-Grunwald-Giemsa stain. The slides were qualitatively and quantitatively evaluated to determine the number of individual nuclear cells per 1000 test cells. The number of cells within the erythrocyte and leukocyte systems was determined and the range of different cells was evaluated. Bone marrow was evaluated in all animals from the control and high dose groups from both the main study and recovery animals.

Animals were euthanized at the end of the administration period for the main study animals and at the end of the additional 21-day recovery period for the satellite groups. All animals underwent a gross examination at the end of the study which included but was not limited to observations of the external body surface and all orifices, all apertures as well as the cranial, thoracic, and abdominal cavities and their contents. Vaginal smears were taken from all females to determine the stage of the estrus cycle. The following organs were weighed; brain, thymus, heart, liver, spleen, kidneys, adrenal glands, testes, epididymides, uterus, ovaries, thyroid with parathyroid, pituitary gland, prostate with the seminal vesicles and coagulating glands. Relative organ weights were also calculated based on the fasting body weight taken prior to euthanasia and based on the absolute weight of the brain. Organ weight results for main study and recovery animals were reported. The following organs and tissues from all animals were preserved for histopathological examinations; brain (cerebrum, cerebellum and medulla/pons), pituitary, spinal cord (cervical, mid-thoracic and lumbar), eyes with optic nerve, Harderian gland, mandibular salivary glands, stomach, small and large intestines including Peyer’s patches if visible (duodenum, jejunum, ileum, cecum, colon and rectum), pancreas, kidneys, adrenal glands, urinary bladder, spleen, heart, aorta, thymus, lymph nodes (mandibular and mesenteric), thyroid with parathyroid, esophagus, trachea, lungs, ovaries with oviducts, uterus with cervix, vagina, testes, epididymides, prostate with seminal vesicles and coagulating glands, skeletal muscle, peripheral nerve (sciatic), femur with joint, skin, mammary gland, bone marrow (sternum), nasal turbinates and all gross lesions. Bones were decalcified, the testes and epididymides were fixed in modified Davidson’s fluid and all others were fixed in 10% formalin. The tissues from main study animals were examined independently by a veterinarian and a pathologist and the results of the pathologist for main study animals are reported in this manuscript. The mandibular and mesenteric lymph nodes, thymus, spleen, lungs, testicles, right kidney, colon, cecum, and jejunum of recovery animals were examined by the veterinarian and no organs from recovery animals were examined by the pathologist.

Histopathological examination of the brain, spinal cord and peripheral nervous system did not show any suspected test material related lesions; therefore, no additional specific nervous system staining was performed. The test material effect on the immune system was evaluated based on results of blood morphology from peripheral blood and bone marrow evaluations, concentration of albumin as an acute phase protein, urea, cholesterol, creatinine, total bilirubin, AST, ALT, ALP, total protein, albumin/globulin ratio, histopathological examination of thymus, spleen, lymph nodes and gut-associated lymphoid tissue if Peyer’s patches were visible and bone marrow, as well as absolute and relative weights of thymus and spleen from all surviving animals.

### Statistical analysis of the maximum tolerated dose and 90-day studies

In the maximum tolerated dose study, the treatment groups were compared to the control group and males and females were analyzed separately. Distribution of the results and homogeneity of variance were checked, in case of normal distribution and homogenous variance, data were analyzed by a one-way analysis of variance (ANOVA) and the Dunnett’s test at p ≤ 0.05. In case of absence of normal distribution or homogenous variance, a non-parametric statistical test was used (i.e., Kruskal-Wallis test). The results are presented in tables in the form of average values and standard deviation.

In the 90-day study, statistical analysis was performed using the following programs: Excel 2007 and STATISTICA 10.0 PL. The treated groups were again compared to the control group and the males and females were analyzed separately. The results are again presented in the form of average values and standard deviation. For the main study animals, the course of the statistical analysis for the main study animals was as follows: initially the normality of the distribution was examined using the Shapiro-Wilk test and the homogeneity of variance by the Brown-Forsythe test, and the results characterized by normal distribution and homogeneous variances were compiled using a one-way analysis of variance followed by Dunnett’s test. In the absence of normality of distribution or non-homogeneous variances, the nonparametric Kruskal-Wallis test was used, followed by Dunnett’s test. The treated recovery group was compared to the recovery control group. The following statistical tests were used to assess intergroup relationships: the normality of the distribution was examined using the Shapiro-Wilk test and the homogeneity of variance by the Brown-Forsythe test. The results which were characterized by normal distribution and homogeneous variances were compiled using the Student’s test. Parameters characterized by non-normal distribution were compiled using Mann-Whitney U test and parameters characterized by normal distribution and non-homogenous variance were compiled by Cochrane-Cox test. Ordinal variables (part of urine parameters) were analyzed using Kruskal-Wallis test followed by Dunnett’s test (main groups) or Mann-Whitney U-test (satellite groups). The weights of internal organs are presented as absolute values as well as relative values (with reference to the body weight and brain weight), in the form of average values and standard deviation. The minimum criterion for statistical significance was determined to be p ≤ 0.05.

## Results

### Reverse bacterial mutation test

Under the conditions of the study, the fold induction over baseline was calculated to determine the mutagenic properties of the test material. The fold inductions in revertant numbers over baseline for all strains tested at all concentrations tested were not considered to be positive because they did not exceed 2.0. The OD_600_ was higher than 2.0 for each overnight culture and ≤0.05 for the negative control which meant that the number of bacteria was suitable for the experiments, the results of the positive and negative controls were within acceptable ranges and the number of positive wells for the positive control were within historical range. The assay met all three of these validity criteria and was therefore considered valid.

The number of reverse mutations for each concentration and strain, with and without metabolic activation, did not exceed the values for spontaneous mutations (S1 Table) and therefore the test material was determined to be non-mutagenic in the bacterial strains used in the study.

### Micronucleus study

No mortalities or abnormal clinical observations were reported in any animals. Some individual animal body weight losses were reported in the males in all groups with the exception of the controls and 250 mg/kg bw dose group and in the females in all groups except the control and cyclophosphamide groups. The body weight losses were all below 5% as compared to the previous measurement with the exception of one male and one female in the mitomycin group and one male and one female in the 250 mg/kg bw group. There was a statistically significant decrease in the numbers of PCE and PCE ratio in the females in the 62.5 and 1000 mg/kg bw groups (p≤0.05). There were no statistically significant changes in the number of MNPCE and frequency of MNPCE in any of the test material dosed animals, as compared to controls (S2 Table). Statistically significant increases in the number of MNPCE and frequency of MNPCE as well as a statistically significant decrease in the number of PCE and PCE ratio were seen in the positive control groups (p≤0.05). The validity criteria for the assay were met and the test material was not cytotoxic to bone marrow in the males based on no changes in the PCE to red blood cell (RBC) ratios at any of the tested doses. In the 1000 mg/kg bw females, the proportion of PCE to RBC decreased approximately 20% as compared to controls which may be attributed to test material exposure. Supercritical Fluid Extract of Aerial Parts of Hemp extract was considered to be negative in this rat bone marrow micronucleus study.

### MTD

No mortalities were reported in the controls or the 1000, 2000 or 2250 mg/kg bw groups, one female was euthanized on the first day of the experiment in the 3000 mg/kg bw group and one female died in the 2500 mg/kg bw group on the second day of the experiment. There were no abnormal clinical signs noted in the control group, however, they were reported in all other groups. In all animals in the 1000, 2000 and 2250 mg/kg bw groups, all abnormal clinical signs were noted on dose day and were then absent by day 2, except for one male in the 2250 mg/kg bw/day group that exhibited porphyrin deposition around the nostrils on day 2 only. In the 3000 mg/kg bw group, the clinical signs were transient in all animals with the exception of one female which showed signs of distress after dosing and was euthanized. In the 2500 mg/kg bw group, the clinical signs were also transient in all animals with the exception of one female which had reduced activity and dejection after dosing on day 1, a distinct decrease in locomotor activity, lethargy, and bristled coat on day 2 and then found dead on day 3.

The body weights and food consumption from all dosed animals were compared to the vehicle controls. In the males, there was a statistically significant decrease in mean body weights on day 1, 2, 3, 4, 5, 7, 13 and 14 in the 1000 mg/kg bw dose group, on days 1, 2, 3, 4, 5, 7 and 14 in the 2000 mg/kg bw group, on days 1 and 2 in the 3000 mg/kg bw group and on days 1, 2, 3, 4, 5, 7 and 14 in the 2500 mg/kg bw group (p≤0.05) (S3 Table). There was a statistically significant increase in the fasted average body weight in the 2250 mg/kg bw group males on dose day, as compared to controls which was not corrected for prior to dosing. In the females, the statistically significant decreases were seen on days 1, 4 and 5 in the 2000 mg/kg bw group and on day 1 in both the 3000 and 2250 mg/kg bw groups (p≤0.05) (S3 Table). There was a statistically significant decrease in mean food intake in the males in all dosed groups on study days 0, 1, 2 and 3, an increased mean food intake on study days 4–7 in the 2000 and 3000 mg/kg bw groups and on study days 8–13 in the 2000, 3000 and 2500 mg/kg bw groups (p≤0.05) (S4 Table). In the females, there was a statistically significant decrease in mean food intake on study days 0–3 in all dose groups and a statistically significant increase on study days 8–13 in the 2000 mg/kg bw group (p≤0.05) (S4 Table).

The results of the hematology, coagulation and clinical chemistry parameters in the dose groups were compared to the results from the control group (S5 and S6 Tables). In the males, the following results were statistically significant from controls; a decrease in the hematocrit value in the 1000, 2000 and 2500 mg/kg bw groups, in the erythrocyte number in the 2000 mg/kg bw group and an increase in the MCHC value in the 1000, 2000 and 2500 mg/kg bw groups while in the females there was a statistically significant decrease in the leukocyte number in all groups except the 2250 mg/kg bw group and a statistically significant decrease in the thrombocyte number in the 2500 mg/kg bw group (all p≤0.05). There were no statistically significant differences in the leukocyte differential evaluation results in the test material-dosed males and females, as compared to controls. In the 1000 mg/kg bw group males, the glucose and cholesterol concentrations were significantly reduced, the sodium concentration was significantly increased in the 2250 mg/kg bw group males and the chloride value was significantly increased in the 1000, 2000 and 2250 mg/kg bw group males (p≤0.05). For the females, there was a significant increase in the albumin/globulin ratio in the 3000 mg/kg bw group and an increase in chloride in the 3000 and 2250 mg/kg bw group (p≤0.05) (S6 Table).

The changes noted at gross macroscopic examination included petechiae or ecchymoses in the thymus, congestion of the thymus and lungs and edema in the lungs, foci of emphysema, foci of atelectasis and were considered to be euthanasia related changes and not related to test material exposure. In the two females that were found dead or euthanized prior to the end of the study, the stomach was very dilated and excessively filled with digestive contents and in the female that was found dead, there was also congestion and dilation of the cecum.

Based on the transient nature of the clinical signs, the absence of toxicologically relevant changes in the hematology and clinical chemistry parameters evaluated, and no abnormal gross macroscopic findings, the maximum tolerated dose of Supercritical Fluid Extract of Aerial Parts of Hemp was determined to be 2250 mg/kg bw. One female animal each in the 2500 and 3000 mg/kg bw groups exhibited adverse clinical signs that resulted in either euthanization or death.

### 90-day oral repeat dose study

One female in the high dose main study group was found dead on day 62 of the study. No abnormal clinical observations were reported in the control and low dose (25 mg/kg bw/day) groups. In the mid dose (90 mg/kg bw/day) animals, transient hyperexcitability was noted in some males and females in the first week of dosing that was not dose dependent. This finding was observed in four males and four females in the main study mid dose group, two males and one female in the main study high dose group, and one male and four females in the high dose recovery group. Thinning of the hair coat on the forearms and abdomen was observed in some males and females from the 4^th^ week of the study. Some abnormal clinical signs were reported in the high dose (324 mg/kg bw/day) group and included transient dejection and a slight decrease in locomotor activity during the first few weeks of the study but not past the 3^rd^ week. Some thinning of the haircoat on the forearms was also noted later in the study. Thinning haircoat and alopecia was seen in one recovery control male. Dejection and a slight decrease in locomotor activity were seen in only the high dose animals. In the high dose recovery group, transient dejection and a slight decrease in locomotor activity was noted in both males and females early in the study, but not past the 4th week of dosing. Thinning of the coat or alopecia on the forearms was reported in both sexes and transient salivation was noted in two high dose recovery males in the 3^rd^ week of the study.

Average body weight data is presented in Tables 1 and 2. No statistically significant differences were found in average body weights in the low or mid dose males as compared to controls while statistically significant decreases in the average body weights in the high dose males was noted in weeks 11.5, 12, 12.5 and 13 of the study, as compared to controls. No changes were noted in the average body weights of the high dose recovery males, as compared to the recovery controls. In the main study females, there were no statistically significant changes in any of the treatment groups, as compared to controls. There were statistically significant decreases in average body weight in the high dose recovery females in weeks 3.5, 6, 9.5, 10, 11.5, 12, 12.5, 13 and 13.5, as compared to recovery control females. The statistically significant lower average body weights in the main study high dose males are attributed to test material exposure but these changes were not seen in the recovery males indicating the effect is reversible. These decreases were not seen in the main study females, however statistically significant decreases in average body weights were seen during the recovery period but by the end of the recovery period, there were no statistically significant differences reported. The decreased average body weight in the high dose males was considered to be related to test material exposure.

**Table 1:** Body weights for the 90-day study—Main and recovery males.

Week	Control	25 mg/kg bw/day	90 mg/kg bw/day	324 mg/kg bw/day	Recovery Controls	Recovery 324 mg/kg bw/day
**Males**						
0	287.5±19.2	287.3±20.3	287.5±13.4	287.5±14.0	294.3±18.0	294.4±19.1
0.5	293.4±19.6	298.5±19.0	296.0±16.0	285.6±13.7	300.9±21.2	292.7±21.2
1	304.1±20.3	307.1±18.8	309.3±18.7	294.7±14.3	314.2±19.7	308.2±20.8
1.5	315.8±22.4	319.6±20.8	319.1±17.6	305.4±13.3	324.6±18.0	316.1±20.9
2	328.9±23.9	332.8±22.2	331.5±18.7	314.5±12.2	336.9±19.1	326.8±25.1
2.5	337.0±23.4	341.8±24.6	340.6±20.4	323.3±13.2	245.5±18.2	339.5±23.0
3	348.4±23.6	352.9±23.8	349.4±23.5	333.6±16.6	355.1±19.5	347.9±25.8
3.5	352.8±20.7	362.7±25.2	358.1±24.6	339.9±16.5	361.4±20.7	354.5±24.7
4	362.9±25.7	364.6±27.1	364.3±25.9	347.5±18.6	367.5±22.5	362.9±27.3
4.5	368.9±24.0	379.0±24.9	372.2±27.6	355.2±18.8	371.9±24.6	369.6±29.6
5	373.7±24.8	387.8±26.0	377.5±27.4	357.4±19.0	379.8±25.9	376.4±31.0
5.5	380.7±24.5	395.2±26.8	383.4±29.5	365.3±20.2	384.8±26.5	379.7±30.3
6	385.7±26.5	397.8±27.2	387.4±29.1	367.5±23.2	389.2±25.3	385.9±32.8
6.5	391.1±26.7	405.8±27.2	393.9±29.6	374.6±23.3	396.5±27.4	392.6±34.2
7	394.4±27.1	406.8±27.2	396.4±28.6	375.0±23.9	401.9±26.8	392.8±34.3
7.5	403.4±28.1	415.3±27.3	402.3±29.5	380.9±23.3	407.1±29.9	398.8±35.6
8	406.7±30.7	421.2±27.1	405.2±31.2	380.4±24.3	411.4±30.2	401.0±34.5
8.5	411.6±28.8	426.3±26.6	409.0±29.4	389.2±24.9	416.4±30.8	409.9±37.7
9	418.8±30.0	433.7±28.3	415.3±30.9	392.1±26.0	423.0±30.0	415.3±36.4
9.5	422.0±30.2	437.5±29.2	415.8±32.4	393.1±26.9	427.8±32.3	416.2±35.1
10	427.5±29.2	440.7±26.5	419.7±31.1	397.2±29.0	432.1±32.2	421.3±39.7
10.5	430.5±28.3	444.2±28.1	422.1±31.7	399.4±30.0	435.7±35.3	424.5±39.3
11	433.1±29.8	446.4±29.6	422.3±31.4	400.7±31.0	436.9±31.6	423.9±37.3
11.5	437.2±29.8	449.8±27.4	425.1±30.9	404.3±31.1*	439.9±34.0	425.1±40.1
12	441.0±28.6	450.9±29.4	427.4±31.4	403.8±30.0*	444.2±35.2	425.8±36.7
12.5	441.7±31.1	454.7±28.0	428.9±31.2	403.8±30.4*	446.0±36.2	426.0±34.7
13	441.5±27.4	456.9±29.1	429.7±32.9	399.6±32.6*	445.9±35.7	423.0±35.7
13^F^	431.5±29.0	442.4±28.5	416.9±33.1	389.2±29.7*	nr	nr
13.5	nr	nr	nr	nr	450.0±35.9	428.1±36.4
14	nr	nr	nr	nr	453.2±35.2	435.8±39.5
14.5	nr	nr	nr	nr	453.4±35.9	442.0±40.5
15	nr	nr	nr	nr	455.3±35.2	441.4±33.8
15.5	nr	nr	nr	nr	461.9±34.2	452.9±36.0
16	nr	nr	nr	nr	455.7±30.7	448.5±36.2
16^F^	nr	nr	nr	nr	443.1±32.6	434.4±36.8

n = 10/sex/group; mean ± standard deviation; F = fasted;

*—statistically significant difference with p≤0.05 Dunnett’s test;

bw = body weight; kg = kilogram; mg = milligrams; nr = not relevant.

**Table 2:** Body weights for the 90-day study—Main and recovery females.

Week	Control	25 mg/kg bw/day	90 mg/kg bw/day	324 mg/kg bw/day	Recovery Controls	Recovery 324 mg/kg bw/day
**Females**						
0	186.6±10.1	186.6±10.3	186.7±10.4	186.8±9.8	207.9±10.2	207.9±10.2
0.5	195.5±10.9	194.3±9.6	187.5±12.4	190.1±8.0	208.6±13.4	203.5±11.0
1	201.9±11.6	198.2±11.8	190.2±11.5	194.1±8.0	213.6±15.2	209.4±10.3
1.5	203.0±13.7	202.2±12.5	199.2±11.5	197.7±7.8	220.2±13.1	210.8±12.1
2	207.1±13.5	206.2±13.6	205.1±10.6	203.4±6.6	222.8±16.9	212.4±12.9
2.5	214.0±14.0	210.9±11.8	209.1±8.0	207.3±8.0	229.6±12.3	222.3±12.4
3	216.3±13.9	214.6±14.4	213.6±8.8	211.6±8.2	231.4±12.5	227.8±11.6
3.5	221.1±13.2	217.3±13.0	215.4±10.0	213.8±8.8	241.5±10.9	229.0±10.8*
4	223.3±12.6	222.2±14.1	220.1±9.8	219.9±11.2	244.0±13.3	234.3±10.2
4.5	228.6±14.1	224.5±15.5	217.1±9.9	222.7±10.7	245.8±16.0	240.5±11.6
5	232.6±13.0	229.8±15.6	221.2±8.3	226.8±11.3	249.8±14.4	244.1±13.0
5.5	232.8±15.8	230.4±16.1	227.3±10.9	228.0±9.3	251.9±13.9	240.5±11.8
6	234.8±15.2	234.5±17.9	230.9±9.6	231.6±8.6	255.4±14.5	243.2±11.0*
6.5	236.1±15.1	236.7±17.8	230.4±7.9	232.2±10.8	257.4±14.8	248.7±13.4
7	239.4±14.1	239.0±17.2	233.5±9.8	232.4±12.4	258.2±12.2	251.0±14.1
7.5	240.8±14.9	240.0±16.2	235.3±9.0	233.5±11.3	263.5±12.7	252.5±15.0
8	242.1±14.7	241.6±17.3	235.8±8.5	236.3±11.2	265.3±14.8	252.8±13.8
8.5	244.5±15.3	244.5±18.2	234.6±9.2	238.6±10.8	266.0±15.8	255.0±13.1
9	245.7±15.5	246.0±18.8	235.0±10.2	238.4±12.9^#^	269.0±16.1	257.1±12.9
9.5	245.5±15.4	245.9±18.0	240.0±11.4	238.4±9.6^#^	269.1±14.5	255.8±12.1*
10	247.2±15.8	246.1±18.6	240.7±10.1	239.4±10.6^#^	270.2±12.8	254.7±14.3*
10.5	248.7±15.2	248.7±18.3	241.2±9.7	239.1±13.8^#^	272.3±13.9	261.1±14.5
11	251.7±16.8	250.2±17.1	242.7±8.3	241.6±13.6^#^	274.2±14.1	262.8±15.5
11.5	251.9±15.6	248.8±17.1	242.9±9.5	242.0±14.3^#^	276.0±14.7	259.7±14.8*
12	251.2±15.8	250.5±17.5	244.1±9.6	242.9±13.8^#^	280.5±15.8	264.2±17.0*
12.5	253.1±16.5	250.7±17.8	240.4±10.9	242.8±13.1^#^	277.6±14.6	260.9±14.3*
13	252.3±17.3	252.1±18.0	243.9±9.0	239.4±13.5^#^	275.9±14.6	259.8±14.0*
13^F^	240.4±16.6	242.2±18.1	233.8±9.3	230.1±13.0^#^	nr	nr
13.5	nr	nr	nr	nr	276.0±13.8	260.1±13.1*
14	nr	nr	nr	nr	279.9±13.7	267.2±16.9
14.5	nr	nr	nr	nr	282.1±12.4	270.4±17.7
15	nr	nr	nr	nr	282.1±13.7	271.4±17.4
15.5	nr	nr	nr	nr	285.0±14.3	276.1+18.4
16	nr	nr	nr	nr	279.3±10.3	271.9+14.5
16^F^	nr	nr	nr	nr	267.1±12.8	260.8+15.0

n = 10/sex/group with the exception of the high dose females where n = 9 from week 9–13 (#); mean ± standard deviation; F = fasted (approximately 15 hours);

*—statistically significant difference with p≤0.05 Dunnett’s test;

bw = body weight; kg = kilograms; mg = milligrams; nr = not relevant.

Food intake was normalized to 100 g body weight (S7 Table). No statistically significant differences were reported in food intake in the main study males and females. In the high dose recovery males, a statistically significant decrease in mean food intake was found during week one but then a statistically significant increase was reported during the final three weeks of the recovery period, as compared to the recovery controls. The findings in recovery males are not considered adverse because there was no effect on body weight. A statistically significant decrease in food intake was reported in the high dose recovery females during much of the main study, with the exception of week 5, 9 and the final four weeks of the recovery period, as compared to recovery controls. These changes are not considered adverse because weights of high dose female animals during the main study and recovery period are within 10% of controls. Further, the effect on food consumption in high dose recovery females during the main study is unique–it did not occur in main study high dose females. It is altogether possible that the results for food intake are artifactual in nature because they are normalized to body weight–reporting as absolute intake would have been more informative because larger animals tend to eat more food than smaller animals.

No test material exposure related adverse effects were noted in the ophthalmological examinations performed prior to dosing or at the end of the study. There were no clear test material exposure related adverse effects observed during the behavioral studies. Changes seen in the open field examinations were reported in both treated and control animals, inconsistent effects were noted with respect to vertical and horizontal locomotor activity and no differences between control and treated animals were seen with the other observations.

There were no statistically significant changes in the hematology parameters evaluated which were clinically or toxicologically relevant as they were either not dose dependent or not accompanied by changes in other related parameters including in the bone marrow evaluation (Table 3). There were a number of statistically significant changes in hematology of the high dose recovery males and females which were not observed in the main study animals. The statistically significant changes noted were considered to be incidental due to the small magnitude of the change, the relatively high value found in the control group, inconsistent findings between males and females, or no supportive changes seen in the bone marrow smears. There were no statistically significant changes in the bone marrow examinations. A statistically significant, prolonged APTT was reported in both the main study high dose males and females, as compared to controls. This change was not seen in the recovery animals and there were no accompanying changes in liver pathology.

**Table 3:** Hematology and coagulation data for the 90-day study—Main study and recovery animals.

Parameter	Control	25 mg/kg bw/day	90 mg/kg bw/day	324 mg/kg bw/day	Recovery Controls	Recovery 324 mg/kg bw/day
**Males**						
RBC (10^12^/L x)	8.92±0.25	9.12±0.38	8.64±0.30	9.05±0.34	8.96±0.33	9.03±0.27
HGB (g/L)	154.20±6.56	155.70±6.15	150.80±4.76	154.67±6.12	157.00±2.05	152.90±3.60*
HCT (1/1) ^†^	0.433±0.019	0.434±0.017	0.422±0.012	0.436±0.017	0.439±0.006	0.427±0.012**
MCV (fL)	48.50±1.41	47.60±1.19	48.85±1.22	48.26±1.12	49.03±1.49	47.27±0.85**
MCH (pg)	17.28±0.56	17.07±0.42	17.47±0.44	17.12±0.33	17.50±0.59	16.94±0.33**
MCHC (g/L)	356.60±4.90	358.80±2.30	357.60±3.06	354.67±3.12	357.40±3.72	358.70±3.13
RET (1/1)^†^	0.011±0.003	0.011±0.003	0.011±0.003	0.009±0.002	0.010±0.003	0.011±0.002
TB (x10^9^/L)	504.10±47.55	447.10±49.77*	469.30±33.41	479.89±43.86	535.00±40.06	534.50±60.91
WBC (x10^9^/L)	2.95±0.40	3.12±0.88	2.76±0.54	2.82±0.69	4.10±0.77	3.00±0.73**
NEU (1/1) ^†^	0.22±0.05	0.21±0.07	0.23±0.05	0.16±0.03	0.20±0.05	0.27±0.09*
LYM (1/1) ^†^	0.78±0.05	0.77±0.07	0.76±0.06	0.83±0.04	0.80±0.06	0.72±0.09*
MON (1/1) ^†^	0.00±0.00	0.00±0.00	0.00±0.00	0.00±0.00	0.00±0.00	0.00±0.00
EOS (1/1) ^†^	0.01±0.01	0.01±0.01	0.01±0.01	0.00±0.01	0.01±0.01	0.00±0.01
Other cells (1/1) ^†^	0.00±0.00	0.00±0.01	0.00±0.00	0.00±0.00	0.00±0.00	0.00±0.00
APTT (s)	29.71±9.17	30.03±10.24	30.01±13.00	47.31±15.55*	34.07±19.24	37.80±16.78
PT (s)	12.61±0.55	12.26±0.89	12.22±0.64	12.63±0.63	12.22±0.74	12.37±0.69
**Females**						
RBC (10^12^/L x)	7.86±0.34	7.76±0.20	8.09±0.35	8.00±0.31	7.92±0.25	7.92±0.26
HGB (g/L)	144.88±7.49	142.33±2.55	146.11±4.62	142.44±6.00	146.30±4.42	146.63±3.50
HCT (1/1) ^†^	0.407±0.022	0.400±0.009	0.410±0.015	0.0405±0.020	0.408±0.013	0.405±0.011
MCV (fL)	51.76±0.93	51.51±1.05	50.73±1.39	50.58±1.42	51.53±1.19	51.11±1.32
MCH (pg)	18.40±0.39	18.36±0.48	18.08±0.46	17.78±0.40*	18.48±0.44	18.51±0.45
MCHC (g/L)	355.63±5.50	356.33±3.77	356.56±5.57	351.89±6.07	358.80±3.39	362.25±4.65
RET (1/1) ^†^	0.009±0.003	0.010±0.002	0.010±0.002	0.010±0.002	0.011±0.002	0.012±0.002
TB (x10^9^/L)	465.25±65.52	444.44±57.77	409.11±89.76	439.11±26.63	487.60±71.11	511.25±74.21
WBC (x10^9^/L)	2.35±0.93	1.83±0.33	2.11±0.50	2.36±0.66	1.92±0.33	2.02±0.61
NEU (1/1) ^†^	0.18±0.08	0.14±0.09	0.15±0.05	0.15±0.08	0.24±0.05	0.19±0.06*
LYM (1/1) ^†^	0.82±0.08	0.85±0.09	0.84±0.06	0.84±0.07	0.75±0.05	0.81±0.06*
MON (1/1) ^†^	0.00±0.00	0.00±0.00	0.00±0.00	0.00±0.00	0.00±0.00	0.00±0.00
EOS (1/1) ^†^	0.00±0.01	0.00±0.01	0.00±0.00	0.01±0.01	0.01±0.01	0.00±0.00
Other cells (1/1) ^†^	0.00±0.00	0.00±0.00	0.00±0.01	0.01±0.01	0.00±0.00	0.00±0.00
APTT (s)	20.78±5.66	21.25±6.96	24.93±6.74	30.11±7.77*	28.46±12.57	24.31±6.64
PT (s)	10.03±0.40	9.93±0.23	10.06±0.46	9.88±0.30	10.71±2.71	9.91±0.25

^†^ = Parameters were reported in this manner in the study report, but the unit was not defined.

n = 10/group with the exception of the main study high dose females which were n = 9. Data are presented as mean ± standard deviation (SD). Statistically significant at *p*≤0.05. APTT = activated partial thromboplastin time; bw = body weight; dL = deciliter; EOS = eosinophils; fL = femtoliters; g = grams; HCT = hematocrit; HGB = hemoglobin; kg = kilogram; L = liters; LYM = lymphocytes; MCH = mean corpuscular hemoglobin; MCHC = mean corpuscular hemoglobin concentration; MCV = mean corpuscular volume; MON = monocytes; mg = milligrams; NEU = neutrophils; pg = picograms; PT = prothrombin time; RBC = erythrocytes; RET = reticulocytes; s = seconds; TB = thrombocytes/platelets; WBC = white blood cells (leukocytes).

Clinical chemistry data for both main study and recovery animals is shown in Table 4. No statistically significant biologically or toxicologically relevant changes were noted in any of the test material dosed animals. The decreases in bile acids seen in the main study treated males were not dose dependent and were within the variability seen in the controls, there were no related histopathology findings, and decreases were not observed in the recovery males. The changes in electrolyte values in the main study males and females were of low magnitude or linked to sample quality and not found in the recovery animals. In the high dose recovery females, two incidental statistically significant findings were reported–a decrease in albumin/globulin ratio and an increase in inorganic phosphorus.

**Table 4:** Clinical chemistry data for the 90-day study main study and recovery animals.

Parameter	Control	25 mg/kg bw/day	90 mg/kg bw/day	324 mg/kg bw/day	Recovery Controls	Recovery 324 mg/kg bw/day
**Males**						
AST (U/L)	150.00±28.25	175.20±72.09	160.50±33.94	162.30±57.17	161.10±35.61	160.90±29.29
ALT (U/L)	43.90±21.35	43.60±16.13	43.40±16.48	50.70±30.49	50.10±10.22	52.30±19.80
ALP (U/L)	116.50±17.41	115.10±22.09	117.90±19.34	112.40±15.78	99.50±25.79	95.80±13.59
A/G Ratio	1.22±0.06	1.22±0.07	1.22±0.05	1.21±0.05	1.11±0.05	1.11±0.04
BILI (μmol/L)	4.28±0.45	5.03±1.33	4.78±0.63	4.65±0.99	4.85±0.53	4.55±0.74
BUN (mmol/L)	5.45±0.53	4.92±0.53	5.25±0.54	5.64±0.74	6.27±1.25	6.22±0.94
CREA (μmol/L)	31.00±4.45	27.00±3.83	30.90±5.22	31.90±5.26	32.30±7.99	27.90±6.94
CHOL (mmol/L)	1.56±0.32	1.52±0.39	1.47±0.26	1.37±0.13	1.98±0.32	1.71±0.26
LDL (mmol/L)	0.37±0.19	0.56±0.29	0.40±0.13	0.46±0.20	0.43±0.06	0.42±0.16
HDL (mmol/L)	1.05±0.21	0.99±0.25	0.93±0.11	0.91±0.08	1.23±0.17	1.09±0.17
BA (μmol/L)	11.44±7.98	5.68±1.84*	5.82±4.23*	4.69±2.94*	4.91±1.72	5.83±1.46
GLUC (mmol/L)	7.34±1.04	7.70±1.03	7.74±0.68	7.98±1.18	8.14±0.98	7.45±0.82
TP (g/L)	60.22±2.17	59.49±2.32	59.35±1.60	60.02±1.45	61.66±2.15	61.53±2.30
ALB (g/L)	33.07±1.18	32.63±1.00	32.59±0.53	32.84±0.71	32.36±1.08	32.27±1.27
GLOB (g/L)	27.15±1.41	26.86±1.67	26.76±1.23	27.18±1.09	29.30±1.40	29.26±1.30
Ca (mmol/L)	2.32±0.05	2.37±0.06	2.34±0.06	2.32±0.06	2.37±0.04	2.33±0.05
P (mmol/L)	1.72±0.17	1.71±0.24	1.89±0.36	1.83±0.27	1.63±0.26	1.65±0.26
Na (mmol/L)	139.30±2.21	140.10±1.66	139.10±2.13	140.40±1.17	143.40±2.17	144.10±0.88
K (mmol/L)	4.02±0.24	4.09±0.13	4.01±0.28	4.13±0.35	4.23±0.25	4.24±0.16
Cl (mmol/L)	101.10±1.45	102.60±1.78	101.50±2.32	103.30±1.77*	103.10±3.03	105.00±1.33
TSH (ng/ml)	2.72±0.48	2.67±0.51	2.96±0.70	2.91±0.58	1.50±0.55	2.18±0.42*
TT3 (ng/ml)	0.64±0.16	0.70±0.17	0.64±0.15	0.71±0.22	0.44±0.20	0.48±0.18
TT4 (ng/ml)	35.71±5.22	35.73±4.07	35.94±5.35	32.98±3.64	34.02±3.92	36.73±3.98
**Females**						
AST (U/L)	136.90±36.35	139.70±31.34	144.30±35.36	159.53±71.90	136.80±34.42	173.60±112.84
ALT (U/L)	34.80±15.79	41.00±12.06	36.40±12.19	49.22±50.45	50.60±26.08	71.00±58.34
A/G Ratio	1.27±0.06	1.24±0.06	1.26±0.05	1.23±0.07	1.18±0.04	1.13±0.05*
ALP (U/L)	54.30±11.28	64.90±8.71	63.90±12.59	47.89±6.90	40.50±7.76	41.30±7.99
BILI (μmol/L)	4.31±0.94	4.83±1.00	4.68±0.88	4.91±1.24	4.58±0.81	5.48±1.88
BUN (mmol/L)	5.95±1.01	5.58±0.74	6.03±1.22	6.39±1.62	7.01±1.40	6.33±0.92
CREA (μmol/L)	35.00±5.40	31.90±5.74	31.10±6.59	35.89±9.74	34.10±5.30	30.80±5.18
CHOL (mmol/L)	1.24±0.39	1.25±0.33	1.19±0.26	1.27±0.32	1.63±0.59	1.65±0.42
LDL (mmol/L)	0.39±0.10	0.44±0.28	0.49±0.21	0.59±0.30	0.41±0.12	0.42±0.17
HDL (mmol/L)	0.85±0.25	0.85±0.23	0.80±0.20	0.86±0.24	1.07±0.36	1.07±0.21
BA (μmol/L)	12.77±8.54	11.34±2.79	12.52±6.17	6.84±1.94	10.12±5.63	10.40±3.18
GLUC (mmol/L)	6.48±0.62	6.58±0.66	6.51±0.65	6.99±0.56	6.50±0.72	6.82±0.95
TP (g/L)	59.26±1.92	61.29±2.89	59.65±2.66	60.67±3.28	64.91±4.52	64.33±4.15
ALB (g/L)	33.13±1.27	33.94±1.33	33.22±1.88	33.43±1.40	35.16±2.19	34.11±1.72
GLOB (g/L)	26.13±1.11	27.35±1.85	26.43±1.01	27.23±2.13	29.75±2.41	30.22±2.51
Ca (mmol/L)	2.36±0.06	2.41±0.06	2.36±0.07	2.36±0.06	2.44±0.08	2.47±0.09
P (mmol/L)	1.63±0.25	1.84±0.27	1.83±0.33	1.86±0.47	1.49±0.22	1.82±0.20*
Na (mmol/L)	142.40±2.37	144.00±1.49	143.50±1.78	143.67±2.06	144.50±1.43	144.30±1.83
K (mmol/L)	3.77±0.27	4.10±0.32	3.84±0.33	4.14±0.34*	3.81±0.40	4.07±0.41
Cl (mmol/L)	104.70±1.25	107.90±1.66*	106.30±1.70	107.33±1.80*	105.60±2.27	106.10±1.85
TSH (ng/ml)	2.92±0.58	3.19±0.52	2.94±0.61	3.03±0.42	2.07±0.77	1.75±0.40
TT3 (ng/ml)	0.64±0.09	0.68±0.26	0.70±0.24	0.63±0.34	0.61±0.18	0.62±0.24
TT4 (ng/ml)	28.98±5.86	26.30±4.73	26.82±3.65	26.72±5.45	23.82±3.44	26.93±3.78

n = 10/group with the exception of the main study high dose females which were n = 9. Data are presented as mean ± standard deviation (SD).

*Significantly different from control, Dunnett’s test (main study) or Student’s t-test (recovery), *p*≤0.05;

A/G Ratio = albumin/globulin ratio; ALB = albumin; ALP = alkaline phosphatase; ALT = alanine aminotransferase; AST = aspartate aminotransferase; BA = bile acids; BILI = total bilirubin; BUN = urea nitrogen; bw = body weight; Ca = calcium; CHOL = cholesterol; Cl = chloride; CREA = creatinine; g = grams; GLOB = globulin; GLUC = glucose; HDL = high density lipoprotein cholesterol; K = potassium; kg = kilogram; L = liters; LDL = low density lipoprotein cholesterol; mg = milligrams; ml = milliliter; mmol = millimoles; Na = sodium; ng = nanograms; P = inorganic phosphorus; TP = total protein; TSH = thyroid stimulating hormone; TT3 –total triiodothyronine; TT4 = thyroxine; U = units; μmol = micromoles.

The value for inorganic phosphorus in the high dose recovery females was similar to the values found at the end of the main study, the value for the recovery controls appeared to be lower than those in the main study, and there were no other correlating clinical chemistry or histopathology findings.

The albumin/globulin ratio was calculated, and no differences were noted in either albumin or globulin and there were no differences noted in the females or the main study animals. There were no biologically or toxicologically relevant changes in any of the urine parameters or in the thyroid hormone parameters evaluated (Table 4) in any of the test material treated animals, as compared to controls.

Multiple gross lesions were reported in all groups which were primarily incidental findings. Excessive fat stores were reported in three main study control males and one mid dose male. The increased fat stores were attributed to exposure to the hemp seed oil vehicle. Emaciation was reported in one main study mid dose male and four high dose males which correlated with the statistically significant decrease in body weight seen at the end of the study. Other lesions were considered to be incidental. There were no apparent adverse effects from test material exposure on the stages of estrus cycle reported for females in both the main and recovery studies.

The absolute organ weights were collected and the relative organ to body weight and relative organ to brain weights calculated (S8–S10 Tables). Organ weights that were affected by administration of the test material are summarized in Table 5. The following were statistically significantly different from controls; absolute liver weight was increased in the high dose females and the relative liver to body weight was increased in high dose males and females; the absolute adrenal weight was increased in the high dose males, the adrenal to body weight was increased in the mid and high dose males, the adrenal to brain weight was increased in the high dose males and weight of the prostate (absolute or relative to brain weight) was decreased in high dose males. No changes were noted in the weights of these organs in the recovery animals. Kidney weights of high dose main study and recovery group males were increased when expressed in terms of body weight only. Other statistically significant findings were reported, however they were considered incidental as they were not dose dependent, occurred sporadically, could be linked to a decrease in body weight or there were no toxicologically relevant histopathological changes. There was a dose-dependent decrease in absolute thymus weight and thymus weight relative to the brain weight in main study males that did not reach statistical significance; however, thymus weights of high dose recovery males were decreased regardless of method of presentation (absolute or relative to body or brain weight).

**Table 5:** Summary of relevant organ weight, macroscopic and histopathological findings.

Organ/tissue	Type of Change Reported	Interpretation
Liver	• Absolute liver weight was increased in the high dose females; relative liver to body weight was increased in the high dose males and females.	No changes were noted in relevant clinical chemistry parameters; the change was not dose dependent and was reversible. Not considered to be adverse.
	• Minimal (3 F;1M) microvascular fatty changes/diffuse seen in the high dose group; not seen in controls or recovery animals	
Adrenal gland	• Increased absolute adrenal weight in the high dose males, adrenal to body weight in the mid and high dose males, and adrenal to brain weight in the high dose males; not seen in recovery animals.	Change in organ weight was reversible
	• Accessory adrenocortical nodule reported in left and/or right adrenal in high dose group (1F, 3M). No examination of adrenal in recovery animals	Nodules not interpreted by study pathologist
Prostate with seminal vesicles with coagulating glands	• Absolute mean weight and relative organ to brain weight were significantly decreased in the high dose male group.	Change in prostate weight was reversible.
	• Diffuse epithelial degeneration, edema and monocyte infiltrations observed in high dose male group (n = 3,6,6, respectively). No examination of prostate in recovery animals	Findings in prostate not interpreted by study pathologist
Kidney	• Increased mean relative kidney to body weights in high dose males and high dose recovery males.	Increase kidney in weight may be an artifact of decreased body weight.
	• No concurrent changes seen histologically and in relevant clinical pathology parameters.	
Thymus	• Absolute weight was not significantly decreased but there was a dose dependent trend for lower weights in main study males. This trend was also noted in the relative organ to brain weight.	Study personnel did not consider the decrease in thymus weight to be related to treatment.
	• The absolute weight, relative organ to body weight and relative organ to brain weight of the thymus in the high dose recovery males were all significantly lower than the corresponding thymus weights in control animals.	Atrophy was not considered to be related to treatment by study veterinarian because it was found in control animals at similar incidence and severity as treated animals. Study pathologist did not comment on this finding.
	• Atrophy was seen in 3 control males and low mid and high dose males (n = 2 2, 3) and 1 control female and low mid and high dose females (n = 4, 4 and 1, respectively). In the recovery animals, atrophy was seen in one female in the high dose group and one male and one female in the control group.	

Numerous histopathological changes were found which were considered to be incidental as they are either commonly found incidental lesions in rats, were found in controls as well as treated animals or were only sporadically reported. Histopathological changes in organs that exhibited changes in weight during the main study and/or recovery period are shown in Table 5. There were no adverse findings in the kidneys and the minimal changes that occurred in the liver were reversible. Histopathological changes were observed in the adrenal glands and prostate (with seminal vesicles with coagulating glands) of some high dose animals, and low incidences of atrophy of the thymus were observed in all groups of animals (including controls). The high dose female that was found dead was examined grossly, and dark red lesions were found near the liver and in the jejunum and colon. This animal also exhibited swollen astrocytes in the hippocampus of the brain, focal hemorrhage in the thymus, lymphocytic hyperplasia in the spleen, degeneration of the renal tubules, cysts in the kidneys and vacuolation of hepatocytes. Study personnel did not attribute any of the changes in the deceased animal to test material administration and mentioned that the changes were likely the result of autolysis; however, because some of the changes in this animal were observed in other animals given the test material, all of the changes cannot be attributed to autolysis. Because the cause of death cannot be attributed to gavage error, relationship to test material administration cannot be discounted.

## Discussion

Results of the safety studies performed on VOHO Hemp Oil (as owned by Verdant Nature LLC) (in collaboration with HempFusion), a supercritical CO_2_ extract from the aerial parts of hemp are described in this manuscript. The test material was not mutagenic in a bacterial reverse mutation assay which tested concentrations up to the limit concentration of 5000 μg/plate (in the presence and absence of metabolic activation). The results of the peripheral blood micronucleus study in the rat show that the test substance is not clastogenic at any dose tested (65.2, 250 or 1000 mg/kg body weight). The maximum tolerated dose in a dose-escalation study was 2250 mg/kg bw. One female was euthanized on the first day of the experiment in the 3000 mg/kg bw group and one female died in the 2500 mg/kg bw group on the second day of the experiment. These animals exhibited decreased activity prior to euthanization or death. Changes in clinical chemistry, hematology and body weight were observed in animals given all doses in the study, so the NOAEL for acute effects was < 1000 mg/kg bw. Based on the results, doses of 25, 90 and 324 mg/kg bw/day were chosen for use in the 90-day study.

In the 90-day study, all animals exposed to the test substance survived to scheduled termination except one high dose female whose death may have been due to administration of the test substance. There were no adverse effects of the test substance on ophthalmoscopic examinations. The study report stated that biologically significant clinical signs were observed at the 90 mg/kg bw/day dose. Clinical findings in the 90 mg/kg bw/day dose group were transient hyperexcitability during the first week only in four males and four females, thinning coat on the forearms of four females, and thinning coat on the abdomen of one female. None of these findings were dose dependent–transient (first week only) hyperexcitability was observed in two males and one female, and thinning coat/alopecia on the forearms were found in five males and no females at the 324 mg/kg bw/day dose. Hair thinning, or alopecia are commonly observed in laboratory rats due to self-barbering precipitated by stress or boredom. In determining whether an effect of a substance is adverse or not adverse, consideration is given to: whether the effect is adaptive or transient, the magnitude of the effect, its association with effects in other related endpoints, whether it is a precursor to a more significant effect, whether it has an effect on the overall function of the organism, if it is a specific effect on an organ or organ system or secondary to general toxicity, or if it is a predictable consequence of the experimental model [21]. As stated in Lewis *et al*. 2002 (20), “it follows that a knowledge of whether or not an effect is reversible may influence significantly the overall interpretation and differentiation of adverse from nonadverse effects”. None of the clinical signs in animals provided the 90 mg/kg bw/day dose are adverse based on these criteria.

The lowest observable adverse effect level (LOAEL) is 325 mg/kg bw/day based on the findings of irreversible effects on behavior of males and females (dejection and decreased locomotor activity), and increased APTT in males and females at this dose. The effect on APTT was reversible; however, it is considered adverse because of the magnitude of the increase and the finding in both sexes. Changes in weights of some organs and/or histopathological changes also occurred in males and/or females given 325 mg/kg bw/day, some of which were irreversible.

Decreased body weights were observed in main study males given this dose; they were considered to be related to administration of the test substance but not adverse because they were within 10% of weights of control males. Further, decreased body weights of high dose recovery males were not observed during the main study or during the recovery period. The reverse situation occurred for high dose females─ main study animals did not exhibit decreases in body weight during the main study, but they were observed in recovery animals during the main study dosing period. The differences in body weight were slight (<10%) and there were no differences in body weight of the high dose recovery females during the 21-day period at the end of the study when the test substance was not administered, therefore the findings were not considered adverse.

Similar to what is observed with other hemp extracts [5, 6], increases in liver weight and fatty changes were observed in high dose animals; however, these changes were not accompanied by increases in ALT, AST, ALP or BIL and did not occur in the recovery group, indicating reversibility. These changes were therefore not considered to be adverse. Relative kidney to body weights were increased in high dose main study males and high dose recovery males, but these changes are likely due to reduced body weights because there were no corresponding changes in pathology or clinical chemistry.

Study personnel did not consider decreased thymus weight to be related to test substance administration. This did not reach statistical significance in main study animals; however, the absolute weight, relative organ to body weight and relative organ to brain weight of the thymus in the high dose recovery males were all significantly lower than the corresponding thymus weights in control animals. The decrease in thymus weight did not appear to be due to atrophy, which was observed at similar incidences and severity in control and high dose males. Because CBD has immunosuppressive effects [22], and the VOHO Hemp Oil preparation used in the study contains 25.2% CBD, it is altogether possible that the decreased thymus weight in high dose males is related to test substance administration.

Changes in adrenal glands such as increased weight, pale appearance and diffuse cytoplasmic vacuolation of the cortical cells of the adrenal glands have been observed in rats given hemp extracts [5, 6]; therefore, the changes in the adrenals observed in the current study are likely related to administration of the test substance. High dose main study males exhibited increased absolute adrenal weight, adrenal to body weight and adrenal to brain weight ratios, and mid dose males exhibited increased adrenal to body weight. These changes were reversible. The increase in adrenal to body weight ratio in mid dose main study males was not considered to be adverse. There was no change in absolute adrenal weight or adrenal to brain weight in the main study mid dose males, and adrenal to brain weight ratio is the optimal index to assess changes in weight of this organ in rats [23]. In contrast to studies with other hemp extracts, increased incidences of cytoplasmic vacuolation of the cortical cells of the adrenals were not observed in this study; however, adrenocortical nodules were reported in left and/or right adrenal in a few high dose animals. Because the adrenal glands were not examined histologically in recovery animals it is unknown whether this effect is reversible. Although the incidence is low, this effect should be considered as possibly related to test substance administration and potentially adverse.

Another finding in the study was decreased absolute mean weight of the prostate with seminal vesicles with coagulating glands and decreased relative weight of these organs to brain weight in the high dose male group. Smaller than normal seminal vesicles and prostate have been observed in male rats given high doses of hemp extracts [6] and should therefore be considered to be related to test substance. Diffuse epithelial degeneration, edema and monocyte infiltrations were also observed in these organs in some high dose males in the current study. Although the changes in weights of the prostate with seminal vesicles with coagulating glands were not observed in recovery animals, pathology of these organs was not examined in this group. Due to lack of evidence of reversibility, the pathological changes observed in the prostate/seminal vesicles/coagulating glands of high dose males should be considered potentially adverse.

In conclusion, the results of the studies described in this manuscript show that the 90-day oral NOAEL and LOAEL for VOHO Hemp Oil are 90 mg/kg bw/day and 321 mg/kg bw/day in male and female Wistar rats. The maximum tolerated dose and the NOAEL for acute effects in these rats are 2250 mg/kg bw and < 1000 mg/kg bw, respectively. The substance is non-genotoxic as analyzed in a bacterial reverse mutation assay and an *in vivo* micronucleus study in Wistar rats.

## Supporting information

S1 Table Reverse mutation assay of VOHO Hemp oil in *Salmonella typhimurium and Escherichia coli*: Mean number ± standard deviation of revertants/plate, fold increase over baseline and binomial B-value. (DOCX)

S2 Table Mammalian erythrocyte micronucleus test bone marrow examination results (mean ± standard deviation). (DOCX)

S3 Table Average body weights for the MTD study. (DOCX)

S4 Table Average food intake for the MTD study. (DOCX)

S5 Table Hematology and coagulation data for the MTD study. (DOCX)

S6 Table Clinical chemistry data for the MTD study. (DOCX)

S7 Table Average food intake [g/100 g b.w./day]—Main study animals and recovery animals. (DOCX)

S8 Table Absolute weight (mean ± standard deviation) of internal organs. (DOCX)

S9 Table Relative organ-to-body weight (mean ± standard deviation) of internal organs (%). (DOCX)

S10 Table Relative organ-to-brain weight of internal organs (%). (DOCX)
