# Supplementary material for: Toxicological safety of VOHO Hemp Oil; a supercritical fluid extract from the aerial parts of hemp
Source: PLoS One. 2021 Dec 31;16(12):e0261900. doi: 10.1371/journal.pone.0261900 (PMC8719773; doi:10.1371/journal.pone.0261900)
Supplement: S1 Table — (DOCX) [file pone.0261900.s001.docx]

**S1 Table**. Reverse mutation assay of VOHO Hemp Oil in *Salmonella typhimurium and Escherichia coli*: mean number ± standard deviation of revertants/plate, fold increase over baseline and binomial B-value

| **Concentration (µg/plate)** | | | **Mean ± SD** | | | **Fold Increase Over Baseline** | | | **B-Value** | | |
| --- | --- | --- | --- | --- | --- | --- | --- | --- | --- | --- | --- |
| **TA 98 (-S9)** | | | | | | | | | | | |
| 0^a^ | | | 3.33 ± 1.15 | | |  | | |  | | |
| 16 | | 2.33 ± 2.08 | | | 0.39 | | | 0.2105 | | |  |
| 50 | | 1.00 ± 1.00 | | | 0.17 | | | 0.0086 | | |  |
| 159 | | 2.00 ± 2.00 | | | 0.33 | | | 0.1212 | | |  |
| 500 | | 0.33 ± 0.58 | | | 0.06 | | | 0.0004 | | |  |
| 1582 | | 1.33 ± 1.15 | | | 0.22 | | | 0.0254 | | |  |
| 5000 | | 1.33 ± 0.58 | | | 0.22 | | | 0.0254 | | |  |
| Positive control | | 48.00 ± 0.00 | | | 8.01****** | | | 1.0000* | | |  |
| **TA 98 (+S9)** | | | | | | | | | | | |
| 0^a^ | | | 2.00 ± 1.73 | | |  | | |  | | |
| 16 | | | 4.67 ± 2.89 | | | 0.84 | | | 0.9855 | | |
| 50 | | | 1.33 ± 1.53 | | | 0.24 | | | 0.0932 | | |
| 159 | | | 2.00 ± 2.00 | | | 0.36 | | | 0.3064 | | |
| 500 | | | 2.33 ± 0.58 | | | 0.42 | | | 0.4490 | | |
| 1582 | | | 1.33 ± 1.15 | | | 0.24 | | | 0.0932 | | |
| 5000 | | | 2.33 ± 0.58 | | | 0.42 | | | 0.4490 | | |
| Positive Control | | | 48.00 ± 0.00 | | | 8.66****** | | | 1.0000***** | | |
| **TA 100 (-S9)** | | | | | | | | | | | |
| 0^a^ | | | 4.67 ± 3.51 | | |  | | |  | | |
| 16 | | | 5.00 ± 0.00 | | | 0.51 | | | 0.2317 | | |
| 50 | | | 6.33 ± 4.04 | | | 0.64 | | | 0.6094 | | |
| 159 | | | 6.00 ± 3.00 | | | 0.61 | | | 0.5124 | | |
| 500 | | | 5.00 ± 2.00 | | | 0.51 | | | 0.2317 | | |
| 1582 | | | 7.67 ± 3.06 | | | 0.78 | | | 0.8910 | | |
| 5000 | | | 3.67 ± 1.53 | | | 0.37 | | | 0.0342 | | |
| Positive Control | | | 48.00 ± 0.00 | | | 4.89****** | | | 1.0000***** | | |
| **TA 100 (+S9)** |  | | |  | | |  | | |  | |
| 0^a^ | | | 6.33 ± 2.08 | | |  | | |  | | |
| 16 | | | 7.00 ± 5.57 | | | 0.69 | | | 0.4191 | | |
| 50 | | | 9.00 ± 2.65 | | | 0.89 | | | 0.8731 | | |
| 159 | | | 5.67 ± 1.15 | | | 0.56 | | | 0.1234 | | |
| 500 | | | 7.67 ± 2.08 | | | 0.76 | | | 0.6004 | | |
| 1582 | | | 7.00 ± 1.73 | | | 0.69 | | | 0.4191 | | |
| 5000 | | | 6.00 ± 1.73 | | | 0.59 | | | 0.1802 | | |
| Positive Control | | | 47.67 ± 0.58 | | | 4.72****** | | | 1.0000***** | | |
|  |  | | |  | | |  | | |  | |
| **TA1535 (-S9)** | | | | | | | | | | | |
| 0^a^ | | | 3.00 ± 3.61 | | |  | | |  | | |
| 16 | | | 3.67 ± 4.73 | | | 0.56 | | | 0.7576 | | |
| 50 | | | 1.00 ± 0.00 | | | 0.15 | | | 0.0126 | | |
| 159 | | | 0.33 ± 0.58 | | | 0.05 | | | 0.0006 | | |
| 500 | | | 1.00 ± 1.00 | | | 0.15 | | | 0.0126 | | |
| 1582 | | | 0.33 ± 0.58 | | | 0.05 | | | 0.0006 | | |
| 5000 | | | 0.33 ± 0.58 | | | 0.05 | | | 0.0006 | | |
| Positive Control | | | 45.00 ± 1.00 | | | 6.82****** | | | 1.0000***** | | |
| **TA1535 (+S9)** | | | | | | | | | | | |
| 0^a^ | | | 0.67 ± 1.15 | | |  | | |  | | |
| 16 | | | 1.67 ± 1.53 | | | 0.33 | | | 0.4423 | | |
| 50 | | | 2.00± 2.00 | | | 0.40 | | | 0.6063 | | |
| 159 | | | 1.33 ± 0.58 | | | 0.26 | | | 0.2794 | | |
| 500 | | | 1.33 ± 0.58 | | | 0.26 | | | 0.2794 | | |
| 1582 | | | 2.00 ± 2.65 | | | 0.40 | | | 0.6063 | | |
| 5000 | | | 1.00 ± 0.00 | | | 0.20 | | | 0.1456 | | |
| Positive Control | | | 39.00 ± 1.00 | | | 7.75****** | | | 1.0000***** | | |
| **TA1537 (-S9)** | | | | | | | | | | | |
| 0^a^ | | | 1.67 ± 0.58 | | |  | | |  | | |
| 16 | | | 0.67 ± 0.58 | | | 0.13 | | | 0.0181 | | |
| 50 | | | 1.00 ± 1.00 | | | 0.19 | | | 0.0546 | | |
| 159 | | | 0.00 ± 0.00 | | | 0.00 | | | 0.0005 | | |
| 500 | | | 1.33 ± 0.58 | | | 0.25 | | | 0.1253 | | |
| 1582 | | | 0.33 ± 0.58 | | | 0.06 | | | 0.0040 | | |
| 5000 | | | 0.00 ± 0.00 | | | 0.00 | | | 0.0005 | | |
| Positive Control | | | 48.00 ± 0.00 | | | 9.16** | | | 1.0000***** | | |
| **TA1537 (+S9)** | | | | | | | | | | | |
| 0^a^ | | | 0.67 ± 0.58 | | |  | | |  | | |
| 16 | | | 0.67 ± 1.15 | | | 0.13 | | | 0.0582 | | |
| 50 | | | 1.00 ± 1.73 | | | 0.20 | | | 0.1456 | | |
| 159 | | | 1.67 ± 0.58 | | | 0.34 | | | 0.4423 | | |
| 500 | | | 0.33 ± 0.58 | | | 0.07 | | | 0.0158 | | |
| 1582 | | | 0.67 ± 0.58 | | | 0.13 | | | 0.0582 | | |
| 5000 | | | 1.00 ± 0.00 | | | 0.20 | | | 0.1456 | | |
| Positive Control | | | 43.00 ± 1.00 | | | 8.66****** | | | 1.0000***** | | |
| ***E. Coli* (-S9)** | | | | | | | | | | | |
| 0^a^ | | | 11.0 ± 2.00 | | |  | | |  | | |
| 16 | | | 10.33 ± 4.73 | | | 0.82 | | | 0.7724 | | |
| 50 | | | 8.33 ± 2.52 | | | 0.66 | | | 0.3049 | | |
| 159 | | | 11.67 ± 4.04 | | | 0.93 | | | 0.9396 | | |
| 500 | | | 13.33 ± 8.62 | | | 1.06 | | | 0.9943***** | | |
| 1582 | | | 9.67 ± 6.66 | | | 0.77 | | | 0.6312 | | |
| 5000 | | | 10.33 ± 7.02 | | | 0.82 | | | 0.7724 | | |
| Positive Control | | | 31.67 ± 1.15 | | | 2.51****** | | | 1.000***** | | |
| ***E. Coli* (+S9)** | | | | | | | | | | | |
| 0^a^ | | | 10.67 ± 1.53 | | |  | | |  | | |
| 16 | | | 15.67 ± 5.51 | | | 1.25 | | | 1.0000***** | | |
| 50 | | | 8.33 ± 6.51 | | | 0.67 | | | 0.4660 | | |
| 159 | | | 9.00 ± 2.65 | | | 0.72 | | | 0.6351 | | |
| 500 | | | 13.67 ± 2.08 | | | 1.09 | | | 0.9992***** | | |
| 1582 | | | 10.67 ± 1.53 | | | 0.85 | | | 0.9176 | | |
| 5000 | | | 8.67 ± 4.93 | | | 0.69 | | | 0.5522 | | |
| Positive Control | | | 25.00 ± 1.00 | | | 2.00****** | | | 1.0000***** | | |
| *Statistically significant difference; B≥0.99 (Binomial B-value)  ** Mutagenic concentration | | | | | | | | | | | |
