# Supplementary material for: Toxicological safety of VOHO Hemp Oil; a supercritical fluid extract from the aerial parts of hemp
Source: PLoS One. 2021 Dec 31;16(12):e0261900. doi: 10.1371/journal.pone.0261900 (PMC8719773; doi:10.1371/journal.pone.0261900)
Supplement: S2 Table — (DOCX) [file pone.0261900.s002.docx]

**S2 Table.** Mammalian Erythrocyte Micronucleus Test Bone Marrow Examination Results (mean ± standard deviation)

| **Parameter** | **Group** | | | | | |
| --- | --- | --- | --- | --- | --- | --- |
|  | **Negative Control** | **Positive Control 1A** | **Positive Control 1B** | **Low Dose**  **62.5 mg/kg bw** | **Medium Dose**  **250 mg/kg bw** | **High Dose**  **1000 mg/kg bw** |
| **Males** | | | | | | |
| **Number of MNPCE** | 4.20 ± 1.48 | 57.00 ± 7.78***** | 42.60 ± 7.50***** | 5.20 ± 2.17 | 6.60 ± 1.82 | 5.00 ± 1.58 |
| **MNPCE frequency (%)** | 0.21 ± 0.07 | 2.85 ± 0.39***** | 2.13 ± 0.38***** | 0.26 ± 0.11 | 0.33 ± 0.09 | 0.25 ± 0.08 |
| **No. of PCE** | 116.8 ± 8.26 | 65.80 ± 7.12***** | 62.4 ± 16.61***** | 106.00 ± 8.15 | 111.00 ± 5.92 | 106.20 ± 4.55 |
| **PCE ratio** | 0.58 ± 0.04 | 0.33 ± 0.04***** | 0.31 ± 0.08***** | 0.53 ± 0.04 | 0.56 ± 0.03 | 0.53 ± 0.02 |
| **Females** | | | | | | |
| **Number of MNPCE** | 5.20 ± 0.84 | 35.40 ± 8.68***** | 42.80 ± 9.96***** | 6.80 ± 1.64 | 5.00 ± 1.87 | 5.20 ± 2.28 |
| **MNPCE frequency (%)** | 0.26 ± 0.04 | 1.77 ± 0.43***** | 2.14 ± 0.50***** | 0.34 ± 0.08 | 0.25 ± 0.09 | 0.26 ± 0.11 |
| **No. of PCE** | 117.60 ± 8.68 | 48.80 ± 4.76***** | 44.80 ± 2.59***** | 105.00 ± 6.28***** | 105.60 ± 12.74 | 94.60 ± 8.44***** |
| **PCE ratio** | 0.59 ± 0.04 | 0.24 ± 0.02***** | 0.22 ± 0.01***** | 0.52 ± 0.03***** | 0.53 ± 0.06 | 0.47 ± 0.04***** |
| n=5 test animals per group; ***** Statistically significant difference with p ≤ 0.05 (Dunnett’s test)  bw = body weight; kg = kilogram; mg = milligrams; MNPCE = micronucleated polychromatic erythrocytes; PCE = polychromatic erythrocytes  Positive Control 1A = cyclophosphamide; Positive Control 1B = mitomycin | | | | | | |
