# Supplementary material for: Toxicological safety of VOHO Hemp Oil; a supercritical fluid extract from the aerial parts of hemp
Source: PLoS One. 2021 Dec 31;16(12):e0261900. doi: 10.1371/journal.pone.0261900 (PMC8719773; doi:10.1371/journal.pone.0261900)
Supplement: S3 Table — (DOCX) [file pone.0261900.s003.docx]

**S3 Table.** Average Body Weights for the MTD Study

| **Day of Experiment** | **Control** | **1000 mg/kg bw/day** | **2000 mg/kg bw/day** | **3000 mg/kg bw/day** | **2500 mg/kg bw/day** | **2250 mg/kg bw/day** |
| --- | --- | --- | --- | --- | --- | --- |
| **Males** | | | | | | |
| 0^f^ | 248.6±24.0 | 229.2±3.7 | 228.8±6.6 | 248.6±5.4 | 239.6±16.0 | 271.0±6.4* |
| 1 | 273.0±27.9 | 240.0±5.0* | 223.6±10.2* | 234.6±7.5* | 234.6±18.1* | 268.0±11.3 |
| 2 | 276.8±25.6 | 242.4±7.0* | 232.6±8.6* | 248.2±10.7* | 239.6±15.4* | 272.0±13.3 |
| 3 | 282.2±26.8 | 245.2±8.4* | 241.2±6.4* | 259.4±7.5 | 249.8±15.2* | 281.4±17.7 |
| 4 | 288.4±27.0 | 248.8±7.9* | 249.2±5.4* | 267.4±7.4 | 255.6±15.0* | 289.0±17.7 |
| 5 | 292.8±29.2 | 256.2±9.2* | 252.4±4.4* | 271.0±6.7 | 260.6±17.5* | 294.8±15.5 |
| 7 | 301.2±28.9 | 261.6±11.5* | 265.2±4.8* | 279.4±9.0 | 270.8±18.1* | 308.0±14.7 |
| 13 | 317.0±28.3 | 281.6±14.4* | 290.2±8.2 | 304.8±8.9 | 291.4±19.4 | 331.4±16.2 |
| 14^f^ | 302.2±28.1 | 270.4±12.7* | 274.2±6.8* | 286.8±6.9 | 274.8±17.7* | 315.6±10.0 |
| **Females** | | | | | | |
| 0^f^ | 169.2±15.0 | 160.4±12.7 | 152.4±4.0 | 162.0±6.8 | 169.2±9.2 | 163.6±7.3 |
| 1 | 184.8±15.6 | 168.2±9.0 | 154.4±9.0* | 160.0±7.7* | 168.2±5.5 | 163.4±10.6* |
| 2 | 187.6±15.2 | 169.4±10.6 | 163.6±5.9 | 172.5±9.1# | 177.8±11.4# | 170.0±10.0 |
| 3 | 188.2±18.3 | 170.0±7.1 | 166.2±4.5 | 171.5±11.1# | 178.5±11.2# | 172.4±12.6 |
| 4 | 188.6±14.9 | 170.6±10.8 | 164.0±5.8* | 174.3±8.4# | 182.0±11.5# | 179.2±11.6 |
| 5 | 194.4±16.3 | 175.8±10.4 | 168.2±5.5* | 179.3±10.7# | 182.3±13.0# | 178.4±5.6 |
| 7 | 197.4±17.5 | 175.8±9.8 | 173.0±4.3 | 179.3±12.7# | 181.5±11.1# | 180.4±15.6 |
| 13 | 205.2±11.7 | 198.2±14.5 | 182.8±5.1 | 190.8±10.8# | 192.0±13.1# | 190.0±9.3 |
| 14^f^ | 211.0±13.9 | 176.2±13.7 | 173.4±5.9 | 178.5±10.3# | 180.8±10.5# | 181.0±9.5 |
| n = 5 animals per group except those noted with # (n=4); f = fasted (approximately 15 hours); data are presented as mean ± standard deviation  * Statistically significant difference with p ≤ 0.05 (Dunnett’s t-test)  bw = body weight; kg = kilogram; mg = milligrams; MTD = maximum tolerated dose | | | | | | |
|  |  |  |  |  |  |  |
