# Supplementary material for: Toxicological safety of VOHO Hemp Oil; a supercritical fluid extract from the aerial parts of hemp
Source: PLoS One. 2021 Dec 31;16(12):e0261900. doi: 10.1371/journal.pone.0261900 (PMC8719773; doi:10.1371/journal.pone.0261900)
Supplement: S4 Table — (DOCX) [file pone.0261900.s004.docx]

**S4 Table.** Average Food Intake for the MTD study

| **Days of Experiment** | **Control** | **1000 mg/kg bw/day** | **2000 mg/kg bw/day** | **3000 mg/kg bw/day#** | **2500 mg/kg bw/day#** | **2250 mg/kg bw/day** |
| --- | --- | --- | --- | --- | --- | --- |
| **Males** | | | | | | |
| 0-3 | 9.6±0.4 | 7.9±0.9* | 6.4±1.0* | 5.8±0.8* | 5.8±0.8* | 6.1±1.3* |
| 4-7 | 8.1±0.3 | 8.1±0.4 | 9.2±0.6* | 9.2±0.2* | 8.8±0.7 | 8.3±0.5 |
| 8-13 | 7.9±0.4 | 7.7±0.1 | 8.7±0.4* | 8.8±0.2* | 8.4±0.5* | 8.0±0.2 |
| **Females** | | | | | | |
| 0-3 | 9.6±0.5 | 7.8±0.6* | 6.5±0.4* | 6.1±0.4* | 6.4±0.9* | 7.5±0.8* |
| 4-7 | 8.8±1.0 | 7.9±0.6 | 9.5±0.2 | 9.5±0.5 | 9.1±0.6 | 8.7±0.5 |
| 8-13 | 8.6±0.6 | 8.4±0.6 | 9.5±0.4* | 9.4±0.6 | 8.9±0.5 | 8.5±0.3 |
| n = 5 animals per group except those noted with # (n=4); data presented as mean ± standard deviation  * Statistically significant difference with p ≤ 0.05 (Dunnett’s t-test)  bw = body weight; kg = kilogram; mg = milligrams; MTD = maximum tolerated dose | | | | | | |
|  |  |  |  |  |  |  |
