# Supplementary material for: Toxicological safety of VOHO Hemp Oil; a supercritical fluid extract from the aerial parts of hemp
Source: PLoS One. 2021 Dec 31;16(12):e0261900. doi: 10.1371/journal.pone.0261900 (PMC8719773; doi:10.1371/journal.pone.0261900)
Supplement: S5 Table — (DOCX) [file pone.0261900.s005.docx]

**S5 Table:** Hematology and coagulation data for the MTD study

| **Parameter** | **Control** | **1000 mg/kg bw/day** | **2000 mg/kg bw/day** | **3000 mg/kg bw/day#** | **2500 mg/kg bw/day#** | **2250 mg/kg bw/day$** |
| --- | --- | --- | --- | --- | --- | --- |
| **Males** | | | | | | |
| HGB (g/L) | 158±7.84 | 154.80±3.27 | 150.00±5.96 | 155.00±3.94 | 153.00±4.53 | 153.20±5.12 |
| HCT (1/1) ^†^ | 0.457±0.025 | 0.429±0.009* | 0.417±0.016* | 0.438±0.009 | 0.428±0.017* | 0.436±0.016 |
| ERY (x 10^12^/L) | 8.79±0.68 | 8.38±0.27 | 7.89±0.30* | 8.65±0.43 | 8.37±0.23 | 8.55±0.31 |
| MCV (fL) | 52.10±1.86 | 51.22±0.81 | 52.86±0.68 | 50.66±1.61 | 51.14±0.97 | 51.00±0.76 |
| MCH (pg) | 18.14±0.86 | 18.48±0.52 | 19.00±0.35 | 17.92±0.67 | 18.26±0.24 | 17.90±0.20 |
| MCHC (g/L) | 348.00±7.75 | 360.40±4.62* | 359.60±2.30* | 353.60±4.77 | 357.00±5.39* | 351.00±2.00 |
| WBC (x10^9^/L) | 6.04±0.71 | 5.28±1.22 | 5.26±1.91 | 3.96±0.61 | 4.78±0.79 | 4.78±1.27 |
| TB (x10^9^/L) | 564.20±40.23 | 551.80±16.59 | 578.00±47.13 | 459.00±133.39 | 494.00±44.41 | 530.00±50.97 |
| RET (1/1) ^†^ | 0.014±0.004 | 0.012±0.002 | 0.015±0.003 | 0.013±0.003 | 0.011±0.002 | 0.012±0.003 |
| NEU (1/1) ^†^ | 0.10 ± 0.04 | 0.14 ± 0.05 | 0.12 ± 0.03 | 0.13 ± 0.03 | 0.11 ± 0.04 | 0.13 ± 0.04 |
| EOS (1/1) ^†^ | 0.00±0.00 | 0.00±0.01 | 0.00±0.00 | 0.00±0.00 | 0.00±0.00 | 0.00±0.01 |
| LYM (1/1) ^†^ | 0.90±0.04 | 0.85±0.06 | 0.88±0.03 | 0.87±0.03 | 0.89±0.03 | 0.86±0.04 |
| MON (1/1) ^†^ | 0.00±0.00 | 0.00±0.00 | 0.00±0.00 | 0.00±0.00 | 0.00±0.00 | 0.00±0.00 |
| Other cells (1/1) ^†^ | 0.00±0.00 | 0.00±0.00 | 0.01±0.01 | 0.00±0.00 | 0.00±0.00 | 0.00±0.00 |
| APTT (s) | 22.98±9.36# | 25.52±10.44 | 17.72±1.63 | 22.66±3.66 | 34.48±19.15 | 22.30±6.50 |
| PT (s) | 11.78±0.56 | 12.30±0.42 | 12.34±0.46 | 12.10±0.48 | 11.92±0.79 | 11.22±0.13 |
| **Females** | | | | | | |
| HGB (g/L) | 149.25±2.99 | 147.60±8.85 | 153.00±3.54 | 151.25±0.96 | 149.50±5.26 | 159.00 |
| HCT (1/1) ^†^ | 0.425±0.008 | 0.416±0.024 | 0.434±0.017 | 0.431±0.010 | 0.428±0.016 | 0.455 |
| ERY (10^12^/L x) | 7.83±0.31 | 8.06±0.55 | 7.95±0.12 | 8.20±0.12 | 8.15±0.21 | 8.75 |
| MCV (fL) | 54.30±1.20 | 51.66±1.91 | 54.56±2.07 | 52.65±0.87 | 52.50±0.81 | 52.00 |
| MCH (pg) | 19.02±0.53 | 18.32±0.65 | 19.22±0.36 | 18.45±0.31 | 18.35±0.24 | 18.20 |
| MCHC (g/L) | 351.50±3.11 | 354.80±2.59 | 353.00±9.30 | 350.75±6.08 | 349.75±1.89 | 349.00 |
| WBC (x10^9^/L) | 5.30±2.00 | 3.14±0.59* | 2.70±0.29* | 2.08±0.56* | 2.72±0.60* | 2.50 |
| TB (x10^9^/L) | 548.50±38.18 | 474.60±31.67 | 503.00±24.44 | 461.25±2.63 | 386.25±134.69* | 436.00 |
| RET (1/1)^†^ | 0.016±0.005 | 0.011±0.003 | 0.012±0.004 | 0.012±0.003 | 0.013±0.002 | 0.009 |
| NEU (1/1) ^†^ | 0.10±0.06 | 0.07±0.03 | 0.11±0.03 | 0.17±0.05 | 0.10±0.05 | 0.11 |
| EOS (1/1) ^†^ | 0.00±0.01 | 0.00±0.00 | 0.00±0.01 | 0.00±0.01 | 0.00±0.00 | 0.01 |
| LYM (1/1) ^†^ | 0.90±0.06 | 0.92±0.03 | 0.88±0.03 | 0.83±0.05 | 0.90±0.05 | 0.88 |
| MON (1/1) ^†^ | 0.00±0.00 | 0.00±0.00 | 0.00±0.00 | 0.00±0.00 | 0.00±0.00 | 0.00 |
| Other cells (1/1) ^†^ | 0.00±0.00 | 0.00±0.00 | 0.00±0.01 | 0.00±0.00 | 0.00±0.00 | 0.00 |
| APTT (s) | 19.85±2.01 | 23.18±7.05 | 18.30±0.71 | 25.40±6.61 | 17.80±1.54 | 32.90 |
| PT (s) | 11.20±0.54 | 11.18±0.30 | 11.64±0.23 | 11.78±0.68 | 11.50±0.71 | 11.00 |

^†^ = Parameters were reported in this manner in the study report, but the unit was not defined.

n = 5/group with the exception of the groups noted with # where n=4 for the females and groups noted with $ where n = 1 for females. Data are presented as mean ± standard deviation where appropriate (SD). Statistically significant at *p*≤0.05 (Dunnett’s test). APTT = activated partial thromboplastin time; bw = body weight; dL = deciliter; EOS = eosinophils; ERY = erythrocytes; fL = femtoliters; g = grams; HCT = hematocrit; HGB = hemoglobin; kg = kilogram; L = liters; LYM = lymphocytes; MCH = mean corpuscular hemoglobin; MCHC = mean corpuscular hemoglobin concentration; MCV = mean corpuscular volume; MON = monocytes; mg = milligrams; MTD = maximum tolerated dose; NEU = neutrophils; pg = picograms; PT = prothrombin time; RBC = erythrocytes; RET = reticulocytes; s = seconds; TB = thrombocytes/platelets; WBC = white blood cells (leukocytes).
