# Supplementary material for: Toxicological safety of VOHO Hemp Oil; a supercritical fluid extract from the aerial parts of hemp
Source: PLoS One. 2021 Dec 31;16(12):e0261900. doi: 10.1371/journal.pone.0261900 (PMC8719773; doi:10.1371/journal.pone.0261900)
Supplement: S6 Table — (DOCX) [file pone.0261900.s006.docx]

**S6 Table:** Clinical chemistry data for the MTD study

| **Parameter** | **Control** | **1000 mg/kg bw/day** | **2000 mg/kg bw/day** | **3000 mg/kg bw/day#** | **2500 mg/kg bw/day#** | **2250 mg/kg bw/day** |
| --- | --- | --- | --- | --- | --- | --- |
| **Males** | | | | | | |
| AST (U/L) | 215.00±122.01 | 162.20±55.03 | 153.20±47.40 | 568.00±627.56 | 257.80±96.39 | 182.20±84.40 |
| ALT (U/L) | 51.80±17.02 | 52.20±21.31 | 50.20±16.75 | 282.40±390.94 | 99.40±44.42 | 65.40±53.64 |
| ALP (U/L) | 188.00±33.47 | 186.00±25.19 | 222.40±29.43 | 211.60±23.96 | 193.40±40.00 | 176.60±46.92 |
| A/G Ratio | 1.27±0.05 | 1.26±0.05 | 1.27±0.06 | 1.27±0.03 | 1.28±0.06 | 1.29±0.08 |
| BILI (µmol/L) | 4.82±0.61 | 4.76±0.53 | 4.52±0.65 | 7.28±4.21 | 5.72±1.88 | 4.04±0.65 |
| BUN (mmol/L) | 6.02±0.56 | 5.16±0.86 | 5.98±0.73 | 6.22±0.57 | 6.12±0.89 | 6.22±0.93 |
| CREA (µmol/L) | 20.00±1.58 | 16.80±2.49 | 25.40±6.50 | 25.60±4.67 | 25.00±7.52 | 26.40±5.94 |
| CHOL (mmol/L) | 2.08±0.13 | 1.58±0.31* | 1.84±0.24 | 2.18±0.13 | 2.06±0.50 | 1.74±0.26 |
| BA (µmol/L) | 30.74±18.88 | 21.06±14.54 | 20.80±8.68 | 19.64±10.35 | 25.22±17.49 | 15.54±5.36 |
| GLUC (mmol/L) | 7.02±1.06 | 5.44±0.50* | 6.50±1.24 | 6.20±0.62 | 7.58±1.09 | 6.10±0.67 |
| TP (g/L) | 57.48±1.86 | 58.68±1.89 | 57.14±3.71 | 59.08±2.28 | 58.38±4.72 | 58.30±2.73 |
| ALB (g/L) | 32.14±1.44 | 32.70±0.97 | 31.94±1.74 | 33.00±1.21 | 32.68±2.22 | 32.78±1.14 |
| GLOB (g/L) | 25.34±0.61 | 25.98±1.16 | 25.20±2.16 | 26.08±1.21 | 25.70±2.59 | 25.52±1.95 |
| Ca (mmol/L) | 2.44±0.05 | 2.53±0.09 | 2.40±0.05 | 2.37±0.08 | 2.44±0.14 | 2.42±0.09 |
| P (mmol/L) | 2.80±0.16 | 2.98±0.23 | 2.54±0.39 | 2.58±0.13 | 2.98±0.37 | 2.64±0.11 |
| Na (mmol/L) | 141.80±1.30 | 144.40±1.95 | 142.40±1.82 | 143.20±1.92 | 143.00±3.24 | 146.20±0.84* |
| K (mmol/L) | 4.32±0.30 | 4.34±0.34 | 4.12±0.30 | 4.58±0.75 | 4.56±0.40 | 4.40±0.35 |
| Cl (mmol/L) | 101.60±0.89 | 106.20±0.84* | 104.40±1.14* | 103.80±1.30 | 102.60±2.30 | 106.40±0.89* |
| **Females** | | | | | | |
| AST (U/L) | 248.60±147.45 | 145.60±42.57 | 140.80±28.23 | 163.75±65.22 | 224.25±262.04 | 473.80±305.93 |
| ALT (U/L) | 59.60±43.19 | 37.60±11.55 | 34.20±8.84 | 37.25±11.24 | 60.50±72.36 | 192.00±173.15 |
| ALP (U/L) | 140.80±30.32 | 106.80±26.51 | 151.80±38.05 | 140.50±13.40 | 116.50±29.78 | 118.60±22.68 |
| A/G Ratio | 1.28±0.05 | 1.27±0.03 | 1.29±0.03 | 1.35±0.02* | 1.33±0.03 | 1.32±0.06 |
| BILI (µmol/L) | 6.84±2.39 | 4.38±0.54 | 4.72±0.74 | 4.03±0.46 | 7.83±7.53 | 9.38±3.23 |
| BUN (mmol/L) | 6.42±1.22 | 6.24±0.36 | 6.68±0.58 | 6.78±0.93 | 6.55±0.95 | 6.66±0.53 |
| CREA (µmol/L) | 28.60±6.54 | 31.20±4.38 | 33.00±3.00 | 27.50±3.11 | 27.00±3.16 | 25.40±5.13 |
| CHOL (mmol/L) | 1.44±0.46 | 1.34±0.21 | 1.04±0.23 | 1.18±0.30 | 1.43±0.46 | 1.42±0.36 |
| BA (µmol/L) | 27.24±15.98 | 16.24±5.96 | 9.50±2.42 | 17.35+16.50 | 30.75+20.87 | 21.06+12.38 |
| GLUC (mmol/L) | 5.52±0.80 | 5.56±0.44 | 5.26±0.34 | 5.50±0.45 | 6.18±0.95 | 5.20±0.67 |
| TP (g/L) | 58.62±1.57 | 59.62±3.31 | 57.60±1.42 | 56.13±1.04 | 58.25±2.59 | 58.26±2.38 |
| ALB (g/L) | 32.90±0.81 | 33.30±1.78 | 32.46±0.77 | 32.25±0.70 | 33.25±1.42 | 33.20±1.76 |
| GLOB (g/L) | 25.72±1.02 | 26.32±1.58 | 25.14±0.80 | 23.88±0.37 | 25.00±1.25 | 25.06±0.84 |
| Ca (mmol/L) | 2.44±0.10 | 2.48±0.04 | 2.42±0.04 | 2.40±0.10 | 2.38±0.09 | 2.41±0.09 |
| P (mmol/L) | 2.70±0.40 | 3.02±0.34 | 3.14±0.21 | 2.65±0.47 | 2.65±0.44 | 2.88±0.16 |
| Na (mmol/L) | 141.80±2.68 | 143.00±2.00 | 143.40±1.52 | 142.75±0.96 | 142.25±2.22 | 143.20±1.10 |
| K (mmol/L) | 4.40±0.75 | 4.30±0.50 | 4.10±0.19 | 4.33±0.32 | 5.00±1.27 | 5.32±0.63 |
| Cl (mmol/L) | 104.00±1.22 | 105.80±1.48 | 104.80±1.64 | 107.75±1.71* | 105.50±1.29 | 108.20±0.84* |

n = 5/group with the exception of the groups noted with # where n=4 for the females. Data are presented as mean ± standard deviation (SD). *Significantly different from control (Dunnett’s test), *p*≤0.05; A/G Ratio = albumin/globulin ratio; ALB = albumin; ALP = alkaline phosphatase; ALT = alanine aminotransferase; AST = aspartate aminotransferase; BA = bile acids; BILI = total bilirubin; BUN = urea nitrogen; bw = body weight; Ca =calcium; CHOL = cholesterol; Cl = chloride; CREA = creatinine; g = grams; GLOB = globulin; GLUC = glucose; HDL = high density lipoprotein cholesterol; K = potassium; kg = kilogram; L = liter; LDL = low density lipoprotein cholesterol; mg = milligrams; mmol = millimoles; MTD = maximum tolerated dose; Na = sodium; P = inorganic phosphorus; TP = total protein; U = units; µmol = micromoles.
