# Supplementary material for: Toxicological safety of VOHO Hemp Oil; a supercritical fluid extract from the aerial parts of hemp
Source: PLoS One. 2021 Dec 31;16(12):e0261900. doi: 10.1371/journal.pone.0261900 (PMC8719773; doi:10.1371/journal.pone.0261900)
Supplement: S7 Table — (DOCX) [file pone.0261900.s007.docx]

**S7 Table**. Average food intake [g/100 g b.w./day] – main study animals and recovery animals

| **Week** | **Control** | **25 mg/kg bw/day** | **90 mg/kg bw/day** | **324 mg/kg bw/day** | **Recovery Controls** | **Recovery 324 mg/kg bw/day** |
| --- | --- | --- | --- | --- | --- | --- |
| **Males** | | | | | | |
| 1 | 7.3 ± 0.9 | 7.5 ± 0.4 | 7.3 ± 0.4 | 6.7 ± 0.7 | 7.7 ± 0.6 | 6.9 ± 0.4* |
| 2 | 6.8 ± 0.5 | 7.0 ± 0.4 | 6.8 ± 0.3 | 6.7 ± 0.7 | 6.8 ± 0.4 | 6.6 ± 0.5 |
| 3 | 6.3 ± 0.3 | 6.4 ± 0.3 | 6.2 ± 0.3 | 6.1 ± 0.2 | 6.4 ± 0.2 | 6.4 ± 0.3 |
| 4 | 5.8 ± 0.4 | 6.1 ± 0.7 | 5.7 ± 0.4 | 5.5 ± 0.3 | 6.1 ± 0.2 | 5.9 ± 0.3 |
| 5 | 5.5 ± 0.3 | 5.7 ± 0.3 | 5.4 ± 0.2 | 5.5 ± 0.2 | 5.7 ± 0.2 | 5.8 ± 0.3 |
| 6 | 5.5 ± 0.3 | 5.6 ± 0.3 | 5.4 ± 0.3 | 5.5 ± 0.2 | 5.5 ± 0.3 | 5.5 ± 0.3 |
| 7 | 5.4 ± 0.5 | 5.5 ± 0.4 | 5.2 ± 0.4 | 5.4 ± 0.3 | 5.4 ± 0.3 | 5.6 ± 0.3 |
| 8 | 5.2 ± 0.5 | 5.3 ± 0.6 | 4.9 ± 0.5 | 5.0 ± 0.3 | 5.4 ± 0.4 | 5.3 ± 0.4 |
| 9 | 4.9 ± 0.4 | 5.0 ± 0.4 | 4.7 ± 0.3 | 4.9 ± 0.2 | 5.1 ± 0.3 | 5.3 ± 0.4 |
| 10 | 4.7 ± 0.5 | 4.8 ± 0.5 | 4.6 ± 0.5 | 4.6 ± 0.4 | 4.8 ± 0.3 | 4.9 ± 0.4 |
| 11 | 4.7 ± 0.4 | 4.6 ± 0.4 | 4.3 ± 0.4 | 4.4 ± 0.4 | 4.8 ± 0.2 | 4.8 ± 0.4 |
| 12 | 4.4 ± 0.3 | 4.4 ± 0.3 | 4.3 ± 0.3 | 4.5 ± 0.2 | 4.5 ± 0.2 | 4.5 ± 0.3 |
| 13 | 4.5 ± 0.4 | 4.3 ± 0.5 | 4.2 ± 0.4 | 4.1 ± 0.3 | 4.7 ± 0.4 | 4.6 ± 0.5 |
| 14 | nr | nr | nr | nr | 5.0 ± 0.4 | 5.5 ± 0.4* |
| 15 | nr | nr | nr | nr | 4.9 ± 0.3 | 5.5 ± 0.4* |
| 16 | nr | nr | nr | nr | 5.2 ± 0.4 | 5.8 ± 0.5* |
| **Females** | | | | | | |
| 1 | 7.6 ± 0.7 | 7.5 ± 0.7 | 7.8 ± 0.6 | 7.1 ± 1.1 | 7.8 ± 0.4 | 6.7 ± 0.6* |
| 2 | 7.2 ± 0.6 | 6.9 ± 0.6 | 7.1 ± 0.5 | 6.6 ± 0.9 | 7.5 ± 0.4 | 6.4 ± 0.6* |
| 3 | 7.1 ± 0.6 | 6.7 ± 0.7 | 6.8 ± 0.6 | 6.9 ± 0.5 | 7.6 ± 0.6 | 6.8 ± 0.3* |
| 4 | 6.5 ± 0.7 | 6.4 ± 0.5 | 6.5 ± 0.5 | 6.7 ± 0.5 | 7.2 ± 0.4 | 6.4 ± 0.4* |
| 5 | 6.0 ± 0.8 | 5.9 ± 0.4 | 6.3 ± 0.6 | 6.2 ± 0.6 | 6.7 ± 0.7 | 6.3 ± 0.5 |
| 6 | 6.2 ± 0.7 | 6.1 ± 0.7 | 6.3 ± 0.6 | 6.3 ± 0.3 | 6.8 ± 0.5 | 6.2 ± 0.5* |
| 7 | 6.1 ± 0.7 | 5.8 ± 0.5 | 5.9 ± 0.6 | 5.8 ± 0.8 | 6.7 ± 0.6 | 6.1 ± 0.4* |
| 8 | 5.9 ± 0.6 | 5.6 ± 0.4 | 5.8 ± 0.6 | 5.8 ± 0.5 | 6.5 ± 0.4 | 5.8 ± 0.6* |
| 9 | 5.5 ± 0.7 | 5.4 ± 0.3 | 5.7 ± 0.6 | 5.7 ± 0.3# | 6.3 ± 0.4 | 6.0 ± 0.9 |
| 10 | 5.7 ± 0.7 | 5.4 ± 0.5 | 5.7 ± 0.6 | 5.6 ± 0.3# | 5.9 ± 0.5 | 5.5 ± 0.4* |
| 11 | 5.6 ± 0.8 | 5.3 ± 0.5 | 5.5 ± 0.7 | 5.5 ± 0.3# | 5.8 ± 0.4 | 5.4 ± 0.4* |
| 12 | 5.1 ± 0.7 | 4.8 ± 0.4 | 5.2 ± 0.5 | 5.2 ± 0.2# | 5.6 ± 0.4 | 5.0 ± 0.3* |
| 13 | 5.4 ± 0.7 | 5.1 ± 0.6 | 5.3 ± 0.7 | 5.6 ± 0.3# | 5.7 ± 0.6 | 5.3 ± 0.5 |
| 14 | nr | nr | nr | nr | 6.0 ± 0.5 | 6.0 ± 0.7 |
| 15 | nr | nr | nr | nr | 6.2 ± 0.4 | 6.5 ± 0.6 |
| 16 | nr | nr | nr | nr | 6.2 ± 0.6 | 6.5 ± 0.4 |
| n = 10 animals per group except those noted with # (n=9)  * Statistically significant difference with p ≤ 0.05 (Student’s t-test)  Bw = body weight; kg = kilogram; mg = milligrams; nr = not relevant | | | | | | |
|  |  |  |  |  |  |  |
