# Supplementary material for: Toxicological safety of VOHO Hemp Oil; a supercritical fluid extract from the aerial parts of hemp
Source: PLoS One. 2021 Dec 31;16(12):e0261900. doi: 10.1371/journal.pone.0261900 (PMC8719773; doi:10.1371/journal.pone.0261900)
Supplement: S8 Table — (DOCX) [file pone.0261900.s008.docx]

**S8 Table**. Absolute weight (mean ± standard deviation) of internal organs

| **Examined Organ** | **Control** | **25 mg/kg bw/day** | **90 mg/kg bw/day** | **324 mg/kg bw/day** | **Recovery Controls** | **Recovery 324 mg/kg bw/day** |
| --- | --- | --- | --- | --- | --- | --- |
| **Males** | | | | | | |
| Brain with cerebellum | 2012.700 ± 122.907 | 2085.400 ± 115.453 | 2079.100 ± 95.504 | 2043.700 ± 78.337 | 2065.500 ± 90.695 | 2115.800 ± 90.360 |
| Pituitary gland | 9.500 ± 2.014 | 9.300 ± 1.829 | 9.700 ± 1.636 | 9.100 ± 2.685 | 11.100 ± 1.792 | 10.500 ± 1.780 |
| Thyroid | 27.300 ± 5.599 | 26.900 ± 3.604 | 27.000 ± 5.164 | 28.100 ± 4.383 | 25.900 ± 4.306 | 24.300 ± 3.860 |
| Thymus | 272.400 ± 88.757 | 270.900 ± 59.592 | 257.800 ± 56.037 | 229.700 ± 43.553 | 278.800 ± 52.302 | 211.500 ± 27.977* |
| Heart | 1017.800 ± 91.591 | 1050.500 ± 101.897 | 1005.800 ± 84.639 | 960.800 ± 80.803 | 1030.000 ± 100.277 | 1088.400 ± 115.644 |
| Liver | 11526.100 ± 1802.472 | 11214.600 ± 1153.647 | 11519.600 ± 1191.535 | 11779.600 ± 1598.532 | 13898.200 ± 1556.609 | 13210.700 ± 1243.732 |
| Spleen | 666.200 ± 72.272 | 641.100 ± 76.973 | 594.700 ± 82.990 | 593.700 ± 92.819 | 665.20 ± 94.561 | 713.500 ± 85.559 |
| Kidneys | 2654.900 ± 281.546 | 2795.400 ± 240.811 | 2735.100 ± 306.747 | 2721.500 ± 289.574 | 2842.900 ± 285.603 | 3047.200 ± 383.847 |
| Adrenal glands | 64.300 ± 5.478 | 65.500 ± 8.370 | 70.100 ± 6.523 | 73.600 ± 8.195* | 71.500 ± 4.170 | 70.200 ± 12.524 |
| Testicles | 3612.500 ± 340.571 | 3509.200 ± 301.596 | 3435.500 ± 266.917 | 3572.800 ± 236.531 | 3639.100 ± 339.581 | 3759.100 ± 324.715 |
| Epididymides | 1400.300 ± 62.333 | 1387.100 ± 126.944 | 1373.000 ± 100.312 | 1347.900 ± 55.417 | 1443.800 ± 97.582 | 1439.400 ± 82.994 |
| Prostate with seminal vesicles & coagulating glands | 2164.200 ± 142.996 | 2083.700 ± 235.960 | 2301.300 ± 216.352 | 1883.800 ± 291.774* | 2300.400 ± 269.550 | 2471.500 ± 309.526 |
| **Females** | | | | | | |
| Brain with cerebellum | 1887.600 ± 125.380 | 1917.800 ± 82.407 | 1904.600 ± 50.746 | 1924.889 ± 69.842 | 1925.800 ± 72.821 | 1931.700 ± 51.694 |
| Pituitary gland | 15.500 ± 2.121 | 14.600 ± 2.591 | 14.900 ± 2.283 | 13.667 ± 3.969 | 17.000 ± 4.028 | 17.000 ± 2.449 |
| Thyroid | 21.900 ± 4.408 | 20.500 ± 2.273 | 18.600 ± 3.627 | 20.222 ± 3.270 | 19.000 ± 2.357 | 22.100 ± 4.630 |
| Thymus | 261.500 ± 74.210 | 273.100 ± 62.605 | 240.700 ± 54.475 | 297.444 ± 46.947 | 257.100 ± 58.274 | 258.100 ± 67.474 |
| Heart | 659.900 ± 35.190 | 627.400 ± 55.879 | 657.900 ± 65.809 | 636.889 ± 45.300 | 730.900 ± 71.216 | 731.100 ± 62.763 |
| Liver | 6822.900 ± 423.417 | 6483.100 ± 477.264 | 6823.300 ± 906.966 | 7608.778± 501.495* | 7781.400 ± 961.206 | 7826.600 ± 866.263 |
| Spleen | 535.900 ± 73.977 | 512.300 ± 51.504 | 477.200 ± 71.425 | 508.333 ± 45.464 | 555.800 ± 46.970 | 603.500 ± 102.318 |
| Kidneys | 1639.800 ± 95.137 | 1696.100 ± 121.716 | 1701.300 ± 127.825 | 1658.333 ± 134.019 | 1876.400 ± 111.120 | 1932.800 ± 145.409 |
| Adrenal glands | 94.800 ± 10.830 | 86.000 ± 11.652 | 87.800 ± 14.958 | 103.556 ± 15.404 | 96.400 ± 8.276 | 99.800 ±11.793 |
| Ovaries | 112.300 ± 25.535 | 115.300 ± 15.041 | 114.200 ± 19.252 | 114.000 ± 16.070 | 131.800 ± 23.437 | 138.900 ± 15.850 |
| Uterus with cervix | 806.700 ± 311.613 | 692.800 ± 260.742 | 681.800 ± 290.574 | 534.778 ± 197.520 | 676.500 ± 380.253 | 561.400 ± 156.374 |
| n = 10 animals per group except 324 mg/kg bw/day females (n=9)  * Statistically significant difference with p ≤ 0.05 (Student’s t-test)  Bw = body weight; kg = kilograms; mg = milligrams | | | | | | |
|  |  |  |  |  |  |  |
