# Supplementary material for: Toxicological safety of VOHO Hemp Oil; a supercritical fluid extract from the aerial parts of hemp
Source: PLoS One. 2021 Dec 31;16(12):e0261900. doi: 10.1371/journal.pone.0261900 (PMC8719773; doi:10.1371/journal.pone.0261900)
Supplement: S9 Table — (DOCX) [file pone.0261900.s009.docx]

**S9 Table**. Relative organ-to-body weight (mean ± standard deviation) of internal organs (%)

| **Examined Organ** | **Control** | **25 mg/kg bw/day** | **90 mg/kg bw/day** | **324 mg/kg bw/day** | **Recovery Controls** | **Recovery 324 mg/kg bw/day** |
| --- | --- | --- | --- | --- | --- | --- |
| **Males** | | | | | | |
| Brain with cerebellum | 0.468 ± 0.042 | 0.472 ± 0.028 | 0.501 ± 0.032 | 0.527± 0.040* | 0.468 ± 0.038 | 0.490 ± 0.040 |
| Pituitary gland | 0.002 ± 0.0005 | 0.002 ± 0.0005 | 0.002 ± 0.0003 | 0.002 ± 0.001 | 0.002 ± 0.0003 | 0.002 ± 0.0003 |
| Thyroid | 0.006 ± 0.001 | 0.006 ± 0.001 | 0.006 ± 0.001 | 0.007 ± 0.001 | 0.006 ± 0.001 | 0.006 ± 0.001 |
| Thymus | 0.063 ± 0.021 | 0.061 ± 0.014 | 0.062 ± 0.010 | 0.059 ± 0.009 | 0.063 ± 0.012 | 0.049 ± 0.004** |
| Heart | 0.236 ± 0.015 | 0.237 ± 0.015 | 0.241 ± 0.012 | 0.247 ± 0.011 | 0.232 ± 0.014 | 0.251 ± 0.017* |
| Liver | 2.667 ± 0.332 | 2.532 ± 0.162 | 2.761 ± 0.133 | 3.018± 0.234* | 3.138 ± 0.278 | 3.041 ± 0.111 |
| Spleen | 0.155 ± 0.018 | 0.145 ± 0.018 | 0.142 ± 0.015 | 0.152 ± 0.017 | 0.151 ± 0.021 | 0.164 ± 0.015 |
| Kidneys | 0.616 ± 0.053 | 0.631 ± 0.028 | 0.655 ± 0.041 | 0.699 ± 0.056* | 0.642 ± 0.049 | 0.701 ± 0.058* |
| Adrenal glands | 0.015 ± 0.001 | 0.015 ± 0.002 | 0.017 ± 0.001* | 0.019 ± 0.002* | 0.016 ± 0.001 | 0.016 ± 0.003 |
| Testicles | 0.839 ± 0.086 | 0.794 ± 0.057 | 0.827 ± 0.067 | 0.923 ± 0.098 | 0.829 ± 0.131 | 0.871 ± 0.107 |
| Epididymides | 0.325 ± 0.017 | 0.314 ± 0.020 | 0.330 ± 0.023 | 0.348 ± 0.024 | 0.327 ± 0.024 | 0.333 ± 0.026 |
| Prostate with seminal vesicles & coagulating glands | 0.503 ± 0.039 | 0.471 ± 0.038 | 0.554 ± 0.055 | 0.484 ± 0.067 | 0.521 ± 0.064 | 0.571 ± 0.068 |
| **Females** | | | | | | |
| Brain with cerebellum | 0.787 ± 0.056 | 0.794 ± 0.039 | 0.815 ± 0.031 | 0.838 ± 0.045 | 0.722 ± 0.028 | 0.742 ± 0.033 |
| Pituitary gland | 0.006 ± 0.001 | 0.006 ± 0.001 | 0.006 ± 0.001 | 0.006 ± 0.002 | 0.006 ± 0.001 | 0.007 ± 0.001 |
| Thyroid | 0.009 ± 0.002 | 0.009 ± 0.001 | 0.008 ± 0.0014 | 0.009 ± 0.001 | 0.007 ± 0.001 | 0.008 ± 0.002 |
| Thymus | 0.109 ± 0.031 | 0.112 ± 0.020 | 0.103 ± 0.023 | 0.130 ± 0.021 | 0.096 ± 0.021 | 0.099 ± 0.026 |
| Heart | 0.275 ± 0.019 | 0.259 ± 0.014 | 0.282 ± 0.027 | 0.277 ± 0.017 | 0.273 ± 0.021 | 0.280 ± 0.012 |
| Liver | 2.846 ± 0.209 | 2.687 ± 0.253 | 2.915 ± 0.338 | 3.309 ± 0.189* | 2.910 ± 0.296 | 2.998 ± 0.246 |
| Spleen | 0.223 ± 0.030 | 0.212 ± 0.020 | 0.204 ± 0.028 | 0.222 ± 0.024 | 0.208 ± 0.020 | 0.231 ± 0.032 |
| Kidneys | 0.685 ± 0.059 | 0.702 ± 0.053 | 0.727 ± 0.041 | 0.722 ± 0.059 | 0.703 ± 0.035 | 0.742 ± 0.053 |
| Adrenal glands | 0.040 ± 0.006 | 0.036 ± 0.005 | 0.038 ± 0.006 | 0.045 ± 0.006 | 0.036 ± 0.004 | 0.038 ± 0.004 |
| Ovaries | 0.047 ± 0.012 | 0.048 ± 0.007 | 0.049 ± 0.008 | 0.050 ± 0.007 | 0.050 ± 0.010 | 0.053 ± 0.006 |
| Uterus with cervix | 0.340 ± 0.141 | 0.290 ± 0.118 | 0.294 ± 0.134 | 0.232 ± 0.083 | 0.256 ± 0.151 | 0.216 ± 0.063 |
| n = 10 animals per group except 324 mg/kg bw/day females (n=9)  * Statistically significant difference with p ≤ 0.05 (Student’s t-test)  ** statistically significant difference with p ≤ 0.05 (Mann-Whitney test)  bw = body weight; kg = kilogram; mg = milligrams | | | | | | |
|  |  |  |  |  |  |  |
