# Supplementary material for: Toxicological safety of VOHO Hemp Oil; a supercritical fluid extract from the aerial parts of hemp
Source: PLoS One. 2021 Dec 31;16(12):e0261900. doi: 10.1371/journal.pone.0261900 (PMC8719773; doi:10.1371/journal.pone.0261900)
Supplement: S10 Table — (DOCX) [file pone.0261900.s010.docx]

**S10 Table.** Relative organ-to-brain weight of internal organs (%)

| **Examined Organ** | **Control** | **25 mg/kg bw/day** | **90 mg/kg bw/day** | **324 mg/kg bw/day** | **Recovery Controls** | **Recovery 324 mg/kg bw/day** |
| --- | --- | --- | --- | --- | --- | --- |
| **Males** | | | | | | |
| Pituitary gland | 0.0005±0.0001 | 0.0004±0.0001 | 0.0005±0.0001 | 0.0004±0.0001 | 0.0005±0.0001 | 0.0005±0.0001 |
| Thyroid | 0.001±0.0003 | 0.001±0.0002 | 0.001±0.0002 | 0.001±0.0002 | 0.001±0.0002 | 0.001±0.0002 |
| Thymus | 0.014±0.004 | 0.013±0.003 | 0.012±0.002 | 0.011±0.002 | 0.014+0.003 | 0.010+0.001* |
| Heart | 0.051±0.006 | 0.050±0.005 | 0.048±0.004 | 0.047±0.004 | 0.050±0.005 | 0.051±0.005 |
| Liver | 0.574±0.091 | 0.539±0.061 | 0.554±0.054 | 0.576±0.074 | 0.675+0.092 | 0.625+0.064 |
| Spleen | 0.033±0.003 | 0.031±0.004 | 0.029±0.004* | 0.029±0.004 | 0.032±0.005 | 0.034±0.005 |
| Kidneys | 0.132±0.016 | 0.134±0.009 | 0.131±0.012 | 0.133±0.014 | 0.138±0.015 | 0.144±0.014 |
| Adrenal glands | 0.003±0.0003 | 0.003±0.0004 | 0.003±0.0003 | 0.004±0.0004* | 0.003±0.0003 | 0.003±0.001 |
| Testicles | 0.180±0.018 | 0.168±0.010 | 0.165±0.013 | 0.175±0.010 | 0.176±0.018 | 0.178±0.011 |
| Epididymides | 0.070±0.004 | 0.067±0.005 | 0.066±0.005 | 0.066±0.002 | 0.070±0.006 | 0.068±0.003 |
| Prostate with seminal vesicles & coagulating glands | 0.108±0.008 | 0.100±0.009 | 0.111±0.012 | 0.092±0.015* | 0.112±0.016 | 0.117±0.015 |
| **Females** | | | | | | |
| Pituitary gland | 0.0008±0.0001 | 0.0008±0.0002 | 0.0008±0.0001 | 0.0007±0.0002 | 0.0009±0.0002 | 0.0009±0.0001 |
| Thyroid | 0.001±0.0002 | 0.001±0.0001 | 0.001±0.0002 | 0.001±0.0002 | 0.001±0.0001 | 0.001±0.0002 |
| Thymus | 0.014±0.004 | 0.014±0.003 | 0.013±0.003 | 0.015±0.002 | 0.013±0.003 | 0.013±0.003 |
| Heart | 0.035±0.002 | 0.033±0.002 | 0.035±0.003 | 0.033±0.002 | 0.038±0.003 | 0.038±0.003 |
| Liver | 0.363±0.029 | 0.339±0.028 | 0.358±0.043 | 0.395±0.017 | 0.404±0.044 | 0.405±0.042 |
| Spleen | 0.029±0.005 | 0.027±0.002 | 0.025±0.004 | 0.026±0.002 | 0.029±0.003 | 0.031±0.005 |
| Kidneys | 0.087±0.006 | 0.089±0.006 | 0.089±0.007 | 0.086±0.006 | 0.098±0.006 | 0.100±0.008 |
| Adrenal glands | 0.005±0.001 | 0.004±0.001 | 0.005±0.001 | 0.005±0.001 | 0.005±0.001 | 0.005±0.001 |
| Ovaries | 0.006±0.001 | 0.006±0.001 | 0.006±0.001 | 0.006±0.001 | 0.007±0.001 | 0.007±0.001 |
| Uterus with cervix | 0.043±0.016 | 0.036±0.014 | 0.036±0.016 | 0.028±0.010 | 0.035±-0.021 | 0.029±0.009 |
| n = 10 animals per group except 324 mg/kg bw/day females (n=9)  * Statistically significant difference with p ≤ 0.05 (Student’s t-test)  bw = body weight; kg = kilogram; mg = milligrams | | | | | | |
|  |  |  |  |  |  |  |
